# Toward a Computational NMR Procedure for Modeling Dipeptide Side-Chain Conformation

**DOI:** 10.1021/acs.jcim.1c00773

**Published:** 2021-11-11

**Authors:** Jesús San Fabián, Ignacio Ema, Salama Omar, Jose Manuel García de la Vega

**Affiliations:** Departamento de Química Física Aplicada, Facultad de Ciencias, Universidad Autónoma de Madrid, 28049 Madrid, Spain

## Abstract

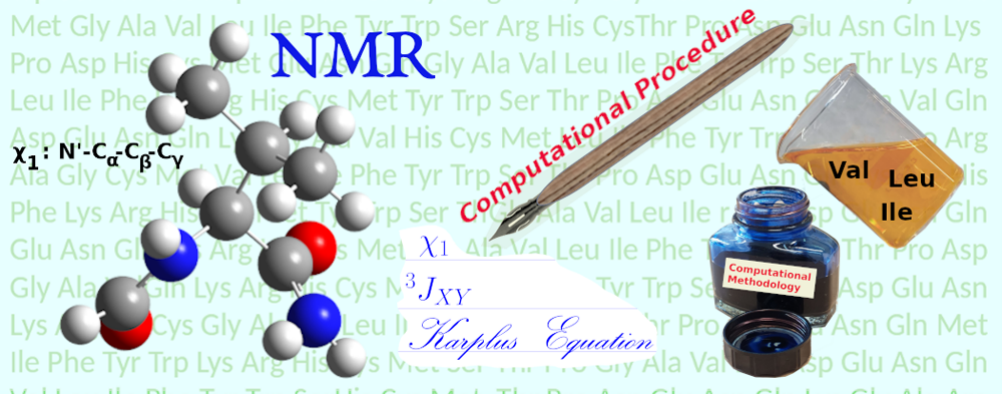

Theoretical relationships between the vicinal spin–spin coupling constants (SSCCs) and the χ_1_ torsion angles have been studied to predict the conformations of protein side chains. An efficient computational procedure is developed to obtain the conformation of dipeptides through theoretical and experimental SSCCs, Karplus equations, and quantum chemistry methods, and it is applied to three aliphatic hydrophobic residues (Val, Leu, and Ile). Three models are proposed: unimodal-static, trimodal-static-stepped, and trimodal-static-trigonal, where the most important factors are incorporated (coupled nuclei, nature and orientation of the substituents, and local geometric properties). Our results are validated by comparison with NMR and X-ray empirical data described in the literature, obtaining successful results on the 29 residues considered. Using out trimodal residue treatment, it is possible to detect and resolve residues with a simple conformation and those with two or three staggered conformers. In four residues, a deeper analysis explains that they do not have a unique conformation and that the population of each conformation plays an important role.

## Introduction

The properties of the amino acid (AA) side chain in proteins are key determinants of protein function, and therefore, for the understanding of life. The diverse chemical nature of the AA side chain is responsible for many specific biochemical functions performed by different proteins.^1^ Side-chain dihedral angles χ_1_ are an important source of information on the dynamics and flexibility of proteins.^2^ Most of these angles correspond to discrete values, and residues generally prefer certain combinations of them.^3^ Side chain χ_1_ is not evenly distributed, but most χ_1_ angles occur around certain values, adopting usually staggered structures.^4^ The most probable side-chain conformations are defined by the statistical analysis of conformational structures.^5^ AA side chains allow for many different types of intramolecular and intermolecular interactions, which are modulated by the dynamics of the side chains.^6,7^ The flexibility and dynamics of AAs, number of conformations that appear per residue, and the frequencies of these conformational changes play an important role in biological properties.

Models are important to reduce the complexity of the protein structure problem. However, a trade-off must be made between complexity and precision. The model must be able to represent different aspects of the structure, and a model will not be useful if it cannot represent a structure close to that of the protein. On the other hand, there are several structural limitations in a protein structure, the variation in bond lengths and angles being small, and the greatest variation occurring in dihedral angles. The prediction of the side-chain structure varies according to the method used. Approaches are different when the protein backbone is unknown than when it is previously and accurately known. Extensive work has been carried out on protein backbone and side-chain modeling^8−10^ Several computational approaches have been developed for the optimization of the side-chain structure in protein design. Most of these methods involve the use of a fixed backbone structure.^11^ This assumption reduces complexity and computational time. Many efficient methods have been developed based on different rotamer libraries and other search methods.^12−14^ These computational approaches were able to predict side-chain torsion angles correctly for proteins. Knowledge of backbone and side-chain conformations have allowed better refinement of experimentally determined structures and enhanced protein design.^15,16^

The direct relationship between protein structure and its functions or properties makes the study of geometry in solution an important issue. Side-chain parameters derived from NMR relaxation experiments in solution display dynamics on the picosecond to nanosecond time scale for AAs and small proteins.^17^ Theoretical calculation and the interpretation of NMR spectra allow the elucidation of chemical structure of biological molecules, particularly when they involve coupled spin systems.^18^ Comparative modeling of protein structures provides high-quality models that are in good agreement with X-ray crystallography or NMR solution structures. In this work, we raise the determination of polypeptide side-chain conformation using theoretical relationships^19^ between vicinal spin–spin coupling constants (SSCCs) and torsion side chain angles χ_1_, that is, the well-known Karplus equations.^20,21^ The relationships between vicinal SSCCs and dihedral angles are formulated by Fourier series which, in turn, are parameterized using accurate theoretical calculations. In order to obtain the Karplus equations, two important sets of data are needed: dihedral angles and SSCCs, ^3^*J*_*XY*_. Vicinal ^3^*J*_*XY*_ couplings depend on the torsional angle, and to a lesser extent on several factors as the substituents attached to the X–C–C–Y fragment and local geometry (bond lengths and angles).^21,22^ As the first approximation, those effects can be considered, at least partially, implicitly included in the Fourier coefficients when they are obtained for a specific AA model fragment. Once these extended Karplus equations have been obtained, they can be used for predicting side-chain conformation by comparison between experimental vicinal SSCCs, ^3^*J*_*XY*_^exp^, and those obtained theoretically for χ_1_ angle, ^3^*J*_*XY*_^teo^(χ_1_).

We extend the earlier work^23^ by considering the findings about basis sets and functionals that predict the best SSCCs and also by incorporating three models. Two of them allowing us to study the rotamers around the side-chain angle. We have applied a computational procedure for determining side-chain dipeptide conformations of three hydrophobic AAs: valine (Val), leucine (Leu), and isoleucine (Ile) in *Desulfovibrio vulgarisFlavodoxin* (strain Hildenborough).^24^ Among all AAs, methyl-containing residues are frequently present in the hydrophobic protein core, and these methyl groups play important roles in protein–ligand foldings and interactions.^25^ There are four aliphatic hydrophobic AAs, the three considered plus Ala, which has been studied previously.^23^ These hydrophobic AAs are nonpolar which implies that they interact weakly with water molecules.^26^ Val is a simple AA with just an isopropyl variable group; Leu has the same variable group as Val but with an extra CH_3_; Ile is an isomer of Leu where the placement of the CH_3_ for a *sec*-butyl rather than an isobutyl side chain. Hydrophobicity increases with the increasing number of C atoms in the hydrocarbon chain. As a consequence, these three AAs are preferentially located inside protein molecules.

This paper is organized as follows. In the section methods, the selection of geometries for its optimization, the method for the SSCC calculations, the Karplus equation fittings, and the three models proposed are described. The computational details are presented next. The results and discussion section is devoted to present and comment on SSCCs, Fourier coefficients, and predicted χ_1_ angles obtained with different theoretical approaches and testing the methodology for Val, Leu, and Ile residues. Finally, the conclusions are presented.

## Methods

**Scheme 1:**
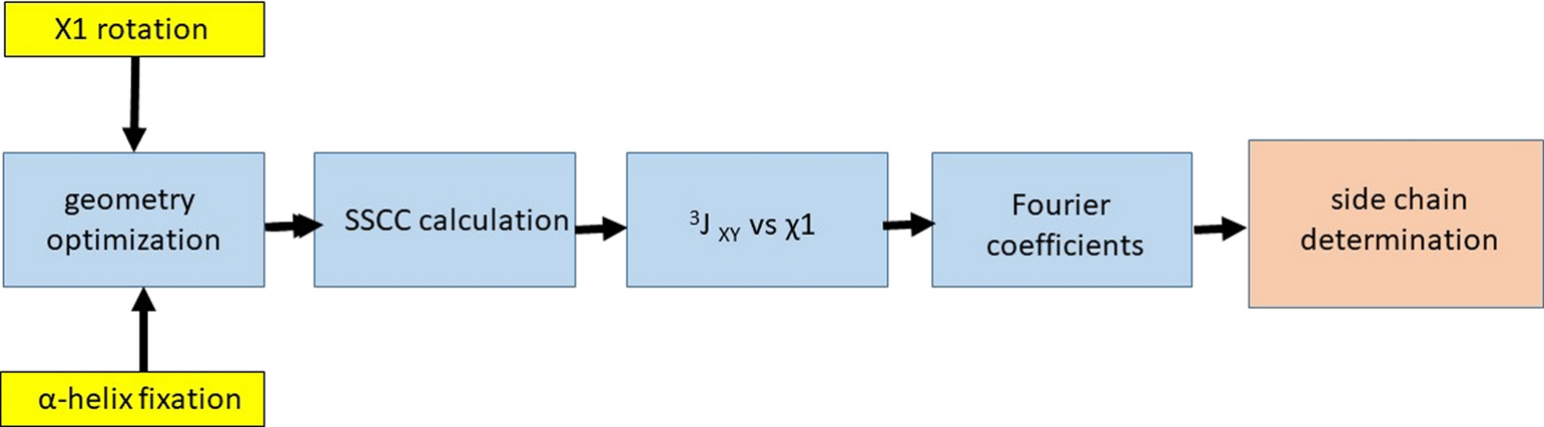
Workflow of the Computational NMR Procedure

The procedure carried out in this work is summarized (Scheme 1) in the following steps:

### Selection and Optimization of Geometries

Most of the AAs have usually favored conformations, that is, those shown in the well-known Ramachandran plots.^15^ The two most important backbone structures are the α conformation (α-helix with ϕ ≈ −64° and ψ ≈ −44°) and the β conformation (β-sheet with ϕ ≈ −121° and ψ ≈ +128°). When AAs are combined to form peptides and proteins, the conformations α, β, and other less populated ones result from steric and noncovalent interactions. When a small dipeptide model, containing only two peptide linkages (−CO–NH−), is used, no interaction appears from further residues and from the surrounding media. Due to the lack of these interactions, a complete geometry optimization of these dipeptide fragments leads to conformations very different from those indicated above (α or β). Thus, the resulting geometries are less realistic and attractive for the study of proteins. Consequently, in this work, the geometry optimizations will be limited to these two main secondary structures, α-helices and β-sheets. The ϕ and ψ angles will be constrained to the respective values indicated above. The effects of these two conformations on the side-chain SSCCs will also be discussed. Clearly, the SSCCs calculated and the Fourier coefficients derived from them will depend on the backbone conformational space. However, the inclusion of this dependence in those coefficients is difficult and complex and we expect, as an approximation, that they will have a minor effect. In a previous work,^23^ a range of differences ca. 15% was found between the SSCCs calculated for α and β-conformations. It should also be noted that the backbone conformational effects are also negligible in the empirical Karplus equations.

**Figure 1:**
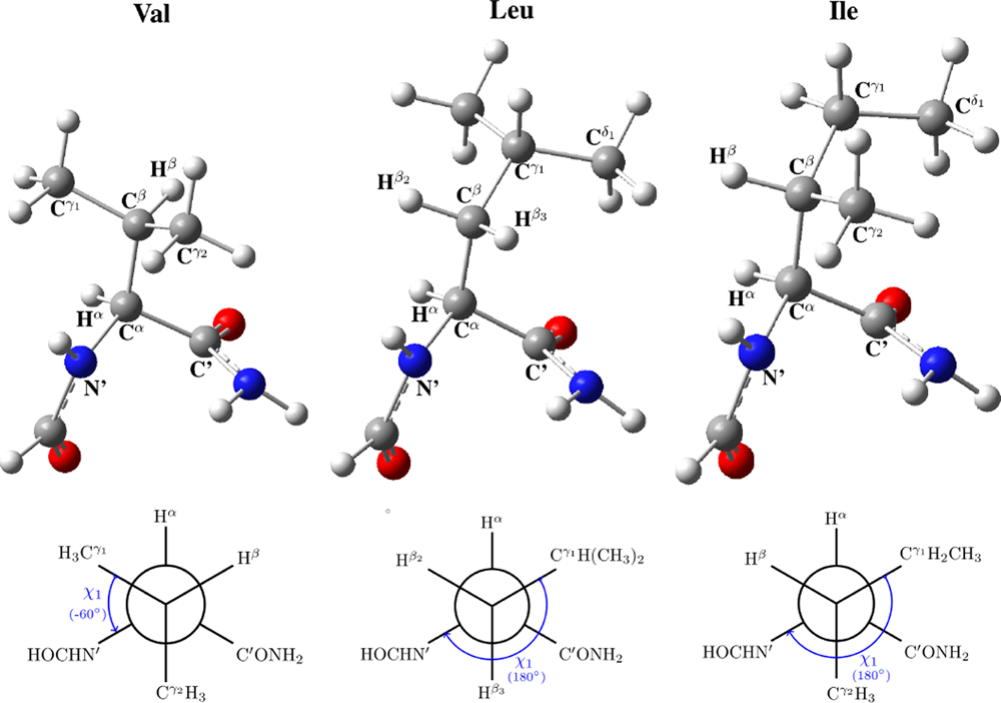
Atoms labels for the definition of χ_1_ (N′–C^α^–C^β^–C^γ_1_^) and χ_2_ (C^α^–C^β^–C^γ_1_^–C^δ_1_^) angles for Val, Leu, and Ile residues and Newman projections defining the χ_1_ angle.

Additionally, the geometries driving the χ_1_ angle were obtained, that is, the parameter χ_1_ = N′–C^α^–C^β^–C^γ_1_^ will be scanned between 0 and 360° at intervals of 30°. Although, as shown earlier,^23^ only those corresponding to the alternated and eclipsed conformations are needed to derive the Karplus equations. For Leu and Ile AAs, the initial χ_2_ angle considered in the geometry optimization (Figure 1) will determine final optimized geometries. Therefore, calculated SSCCs, will also be affected, although in a small magnitude, by this χ_2_ orientation.

### SSCC Calculations

Once the geometries, with the indicated restrictions, are obtained we need to calculate the vicinal SSCCs involved around the χ_1_ angle. For Val, Leu, and Ile AAs, nine vicinal SSCCs can be calculated around the C^α^–C^β^ bond, which are of six different types: ^3^*J*_H^α^,H^β^_, ^3^*J*_H^α^,C^γ^_, ^3^*J*_C′,H^β^_, ^3^*J*_C′,C^γ^_, ^3^*J*_N′,H^β^_, and ^3^*J*_N′,C^γ^_.

The most accurate way to calculate these couplings is by using wave function (WF) methods that have proved to give reliable results on small molecules.^27−29^ However, owing to the size of the AA fragments and the large amount of SSCCs needed, we consider combining them with methods based on density functional theory (DFT).^23,28^

Within DFT, SSCCs depend not only on the used basis set, as in WF methods, but also on the utilized functional. Therefore, DFT calculations must be tested in specific cases to find the best basis set and functional for these AAs and sometimes for each type of SSCCs. These tests will be carried out by comparing DFT results with those obtained with WF calculations and also by comparing the final χ_1_ angles with those obtained from NMR and X-ray measurements.

### ^3^*J*_*XY*_ versus χ_1_ and Karplus Equations

Once the calculated SSCCs and dihedral angles are available, the Fourier coefficients for the different vicinal SSCCs are obtained by means of single least-squares regression. These sets of coefficients will be compared with those obtained empirically by NMR.^30,31^ From a set of six values of ^3^*J*_*XY*_ and dihedral angles, it is possible to obtain up to six Fourier coefficients *C*_0_, *C*_1_, *C*_2_, *C*_3_, *S*_1_, and *S*_2_ for the following extended Karplus equation
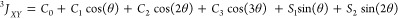
1where θ is the dihedral angle between the coupled nuclei. In order to analyze and compare the calculated results, that is, to say, the different sets of six SSCCs or Fourier coefficients obtained with two different approaches (set1 and set2), we will use the following root-mean-squared deviation (rmsd) statistical parameter^23^
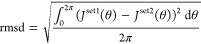
2Within this definition, the rmsd between two different sets of Fourier coefficients can be written
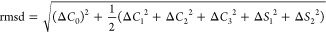
3where Δ*K*_*n*_ = *K*_*n*_^set1^ – *K*_*n*_^set2^ with *K* = *C* or *S* and *n* = 0, 1, 2, ...

To compare results obtained with different theoretical methods and basis sets, it is convenient to combine the rmsd values into a relative value that incorporates the nine studied SSCCs. Thus, the following average weighted rmsd (awrmsd) values (in %) are defined. The relative weights correspond to the average couplings taken as the respective |*C*_0_| values.
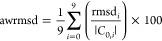
4where rmsd_*i*_ are the values obtained using eq 2 or 3 for each type *i* of SSCCs. The values taken for *C*_0,*i*_ are those calculated at the SOPPA(CCSD)/aug-cc-pVTZ-J.

### Models for Side-Chain Dihedral Angle χ_1_

When the Karplus equations are established, we can use them in combination with experimental ^3^*J*_*XY*_^exp^ to find out the χ_1_ dihedral angle. In this work, we have developed three models:

(i) Unimodal-static (UMS): In this first model, the χ_1_ angle is determined considering the existence of a single conformer and minimizing for each residue (res) the following rmsd function

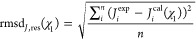
5where *n* is the number of experimental *J*_*i*_^exp^ couplings for a given residue.

This model usually predicts two different minima, if one is at χ_1_, the other will be around χ_1_ + 180°.^32^ This ambiguity is inherent in the degeneration of the Karplus equation that even with up to nine experimental couplings gives a multi-valued solution.^32^ To avoid this ambiguity, we consider within this model the results that fulfill two conditions: (i) the determined χ_1_ value corresponds to an staggered conformer within an uncertainty of ±30°, and (ii) the population for this unimodal conformer, calculated as suggested below [trimodal-static-staggered (TMSS)], is larger than 60%.

(ii) TMSS: This second model considers three staggered conformers with χ_1_ at 60, 180, and −60°, and the populations will be determined by minimization of the following equation

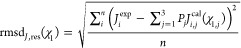
6where populations *P*_*j*_ are calculated using the Quadprog R package^33^ to minimize the rmsd_*J*,res_(χ_1_), eq 6, with the conditions: *P*_60_ + *P*_180_ + *P*_–60_ = 1 and *P*_*i*_ ≥ 0.^34,35^ In this model, two parameters are determine, that is, the population of two conformers.

(iii) Trimodal-static-trigonal (TMST): The third model considers also a trigonal symmetry, but now three parameters are found by minimization: two populations and one χ_1_ angle. The other two angles are considered χ_1_ ± 120° following a trigonal symmetry. Although this trigonal symmetry does not have to be fulfilled, it is likely that the χ_1_ angle for the most populated conformer is reasonable. In this model, the rmsd function will be determined by the following equation

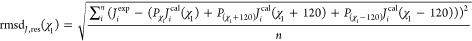
7

### Analysis of the Results

The obtained torsional χ_1_ angles will be compared with three sets of empirical values. The first two sets are those derived by Pérez et al.^30^ and Schmidt et al.^31^ using empirical NMR SSCCs and Karplus equations. The third set corresponds to the average X-ray torsional angles derived from high-resolution X-ray structures. Schmidt et al.^31^ made a selection of calculated torsion angles obtained from eight different X-ray entries within the PDB.^31^ This X-ray set has been extended with five more recent data obtained from PDB entries^36^ (Table S1, Supporting Information). In the present work, X-ray reference values are obtained averaging the X-ray torsional angles after removing the outlier torsion angles, that is, those that deviate more than 30° from the average. Thus, χ_1_ values obtained when the protein crystallizes in different and minority conformations are not considered, at least in a single conformer model. Some removed X-ray angles can be interpreted on the basis of torsion angle dynamics and localization uncertainties.^31^

Besides, the above indicated rmsd_*J*,res_(χ_1_) values, eqs 5 to 7, the following two statistical parameters are considered. First
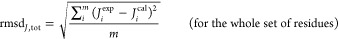
8compares the whole set of experimental *J*^exp^ SSCCs (*m* values) with those calculated for the predicted χ_1_ values. Second
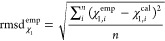
9compares the empirical χ_1_ angles with those predicted in this work. χ_1,*i*_^emp^ is the dihedral angle empirically obtained by Schmidt^31^ and Pérez,^30^ or the above indicated X-ray average angles; χ_1,*i*_^cal^ corresponds to the values calculated in this work, and *n* is number of values included in the statistics, taking into account that the values with discrepancies larger than 40° are not considered.

In order to obtain the needed distance between two angles and the average, the following circular statistic approach^37^ was used: the angle, here χ_1,*i*_, is represented by its equivalent vector (*x*_*i*_ = cos χ_1,*I*_ and *y*_*i*_ = sin χ_1,*i*_). The distance between two angles (χ_1,*i*_ and χ_1,*j*_), that is, the minor angle between them, is calculated by
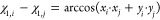
10

The average ⟨χ_1_⟩ angle is calculated as
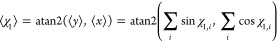
11where atan2 is the four quadrant inverse tangent function returning an angle between −π and π.

## Computational Details

In the present work, three different dipeptides have been studied corresponding to Val, Leu, and Ile residues. Molecular models and the definition of atoms are shown in Figure 1. These dipeptide models present a reliable size for the computational calculations and incorporate the main effects on the side-chain SSCCs except those of large range, for instance, noncovalent interaction effects. In our previous work on Ala side-chain SSCCs, the appropriate Fourier series, the best quality/cost WF and DFT approaches, and basis set for use on other AAs were established.^23^

Partial optimized geometries have been carried out at the B3LYP/6-31G(d,p) level^38−42^ using the Gaussian program.^43^ Two sets of geometries were optimized, one with α-helix and another with β-sheet backbone conformations, respectively, where the ϕ and ψ angles were constrained to −64 and −44° for α and to −121 and 128° for β-conformer. The χ_1_ angle was kept frozen for the three molecules between 0 and 330° at intervals of 30°. These 12 resulting values will be used to analyze the geometry, although selecting six values of χ_1_ (0, 60, 120, 180, −120 and −60°) for the SSCCs calculations. In addition, the angle χ_2_ was positioned in Leu and Ile at the three staggered conformations before the optimization, and it was checked that the angle after the optimization remains around the initial staggered position. Results presented in this work for SSCCs or Karplus equations for Leu and Ile will correspond to the average between the three χ_2_ conformers.

SSCCs have been calculated using WF and DFT methods. The WF ones will be carried out at the limit of our computational resources, using the DALTON suite program.^44,45^ The level of theory chosen is based on our previous results for Ala.^23^ The WF method is the SOPPA(CCSD)^46^ which considers the electron correlation efficiently with a reasonable computational cost for these molecules.^23^ The selected functionals were B3LYP, B972, wB97X, wB97XD, and S55VWN5, which give the best results for Ala.^23^ The basis sets used in these calculations were the 6-311++G**-J^47^ and the aug-cc-pVTZ-J,^48^ both developed specifically to calculate SSCCs. The last and larger basis set was used preferably in the more cost-efficient DFT calculations.

## Results and Discussion

### Geometries and χ_2_ Rotamers

**Figure 2:**
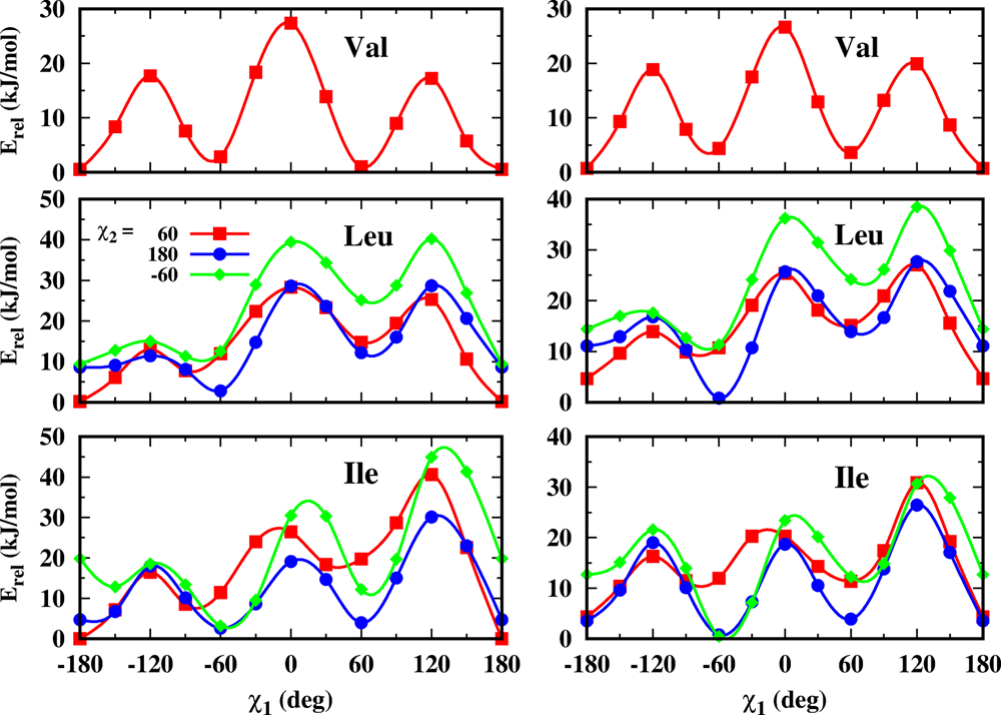
Energy profiles for Val, Leu, and Ile vs χ_1_ angle for α-helix and β-sheet conformations. For Leu and Ile, the three staggered χ_2_ conformers are shown. Energy profiles were obtained at the B3LYP/6-31G(d,p) level.

The profiles of energies are obtained from the geometries optimized fixing the χ_1_ angle (Figure 2). Leu and Ile dipeptides present three different staggered conformers around χ_2_ angle which are also included in Figure 2.

In protein studies, the dihedral angles θ between the substituents around the C^α^–C^β^ bond are related to χ_1_ (N′–C^α^–C^β^–H) by the equation θ = χ_1_ + Δθ, where the phase shift Δθ is usually taken as 0, 120, or −120° (denoted here as Δθ^tetrah^).^30^ However, as previously detected in Ala,^23^ the optimized geometries show systematic deviations between the dihedral angles optimized and those calculated using the above relationship. These deviations, θ^calc^ – (χ_1_ + Δθ^tetrah^), average up to 15° over the calculated geometries when the χ_1_ angle is driven (Table S2, Supporting Information). In order to improve the Karplus equation accuracy, or at least to reduce the systematic errors, the new Δθ^proposed^ could be used instead of the tetrahedral ones.

### Fourier Coefficients

Fourier coefficient for Val, Leu, and Ile dipeptides calculated at high-level SOPPA(CCSD)/6-311++G**-J are presented in Table S3 (Supporting Information). For Leu and Ile residues, the presented coefficients are those corresponding to the average between the results for the three staggered conformers around χ_2_ (Figure 1). Fourier coefficients calculated with DFT methods, for α- and β-conformers, as well as the empirically derived coefficients by Schmidt et al.^31^ and Pérez et al.^30^ are also available in Tables S4–S7 (Supporting Information).

**Figure 3:**
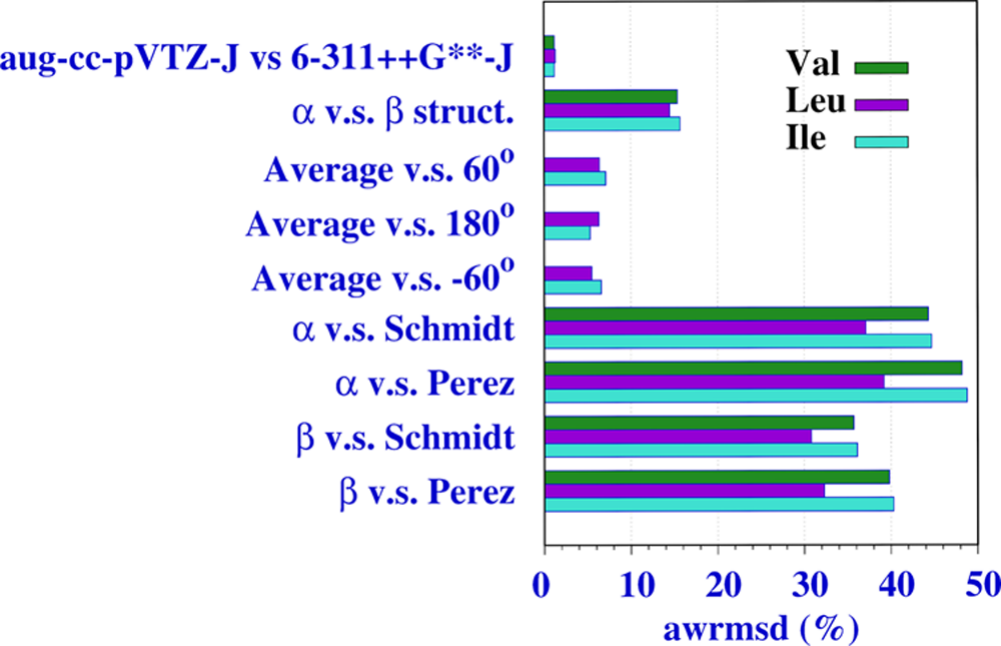
Values of awrmsd (%) for Val, Leu, and Ile between pair of results.

The comparison of the Fourier coefficients calculated with different approaches is presented briefly in Figure 3 and in detail in Table S8 (Supporting Information). First, we evaluate those obtained with the 6-311++G**-J and aug-cc-pVTZ-J basis sets, both calculated using the B3LYP functional. The former basis set was used in WF calculations owing to its smaller size, while the larger aug-cc-pVTZ-J basis set was employed in most DFT calculations because these methods are computationally more cost-effective. The rmsd and awrmsd values show that the differences between the results of both basis sets are negligible. Only SSCCs between protons have a rmsd greater than 0.1 Hz (around 0.19 Hz). For the remaining couplings, rmsd values are smaller than 0.05 Hz. The awrmsd values are around 1.2% for the three AAs. Only the α-conformer results are shown due to the similarity with those of β. Second entry compares results for α- and β-conformers. Both calculated at the SOPPA(CCSD)/6-311++G**-J level. For the three AAs, the awrmsd values are around 15% which is a small but not negligible amount. Considering the rmsd, we found values up to 0.86 Hz for proton–proton SSCCs, the largest ones. For Leu and Ile, entries 3–5 compare the Fourier coefficients for each of the three χ_2_ staggered conformers with those obtained as an average of the three conformers. Values for these comparisons were also calculated at the highest SOPPA(CCSD)/6-311++G**-J level. The awrmsd values, similar for both AAs, range between 5 and 7%, and the largest rmsd (0.4 Hz) is that of  in Leu.

Fourier coefficients calculated at the SOPPA(CCSD)/6-311++G**-J level are compared in the last four entries with those empirically obtained by Schmidt^31^ and Pérez^30^ (Table S7, Supporting Information). The differences observed are large. Several reasons justify those results: (i) *C*_3_, *S*_2_, or *S*_1_ coefficients are not considered in Schmidt’s (the first two) or Pérez’s (the three) results; (ii) *C*_1_ coefficients are forced in the empirical determinations to be negative except for nitrogen involved SSCCs which are forced to be positive, owing to the change of sign in the ^15^N magnetogyric ratio; and (iii) coefficients for the same type of couplings, for instance,  and , are considered to be equal, thus neglecting the effects of the substituent position on those SSCCs.^22^ Coefficients for Leu have a lower awrmsd, and those obtained for the β conformer are more similar to those obtained empirically than that in the α conformer.

**Figure 4:**
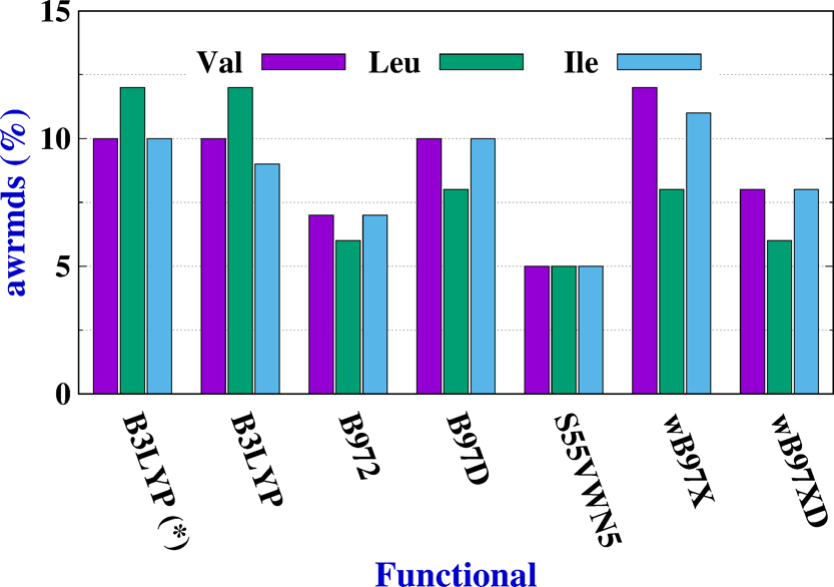
Values of awrmsd (%) for Val, Leu, and Ile when SOPPA(CCSD)/6-311++G**-J results are compared to DFT ones. Only α-conformer results are shown. The aug-cc-pVTZ-J basis set was used, except for (*) where the 6-311++G**-J was employed.

The performance of DFT methods is analyzed considering the results, as shown in Figure 4 and Table S9. In this Figure 4, the awrmsd between the SOPPA(CCSD) and DFT Fourier coefficients are presented. As indicated above, functionals were selected from those that yielded the best SSCCs when compared with WF values.^23^ Therefore, it is not surprising that the results present awrmsd values that are smaller than 12%. The best results are those of the S55VWN5 functional (5% awrmsd) followed by those of B972 and wB97XD functionals (8% awrmsd) for all three AAs.

### Optimizing Side-Chain Torsion Angle χ_1_

Dihedral angle χ_1_, rotamer populations, and statistical parameters calculated using the procedures indicated above are presented and compared to the results previously obtained by NMR and the average X-ray values.^30,31^

**Figure 5:**
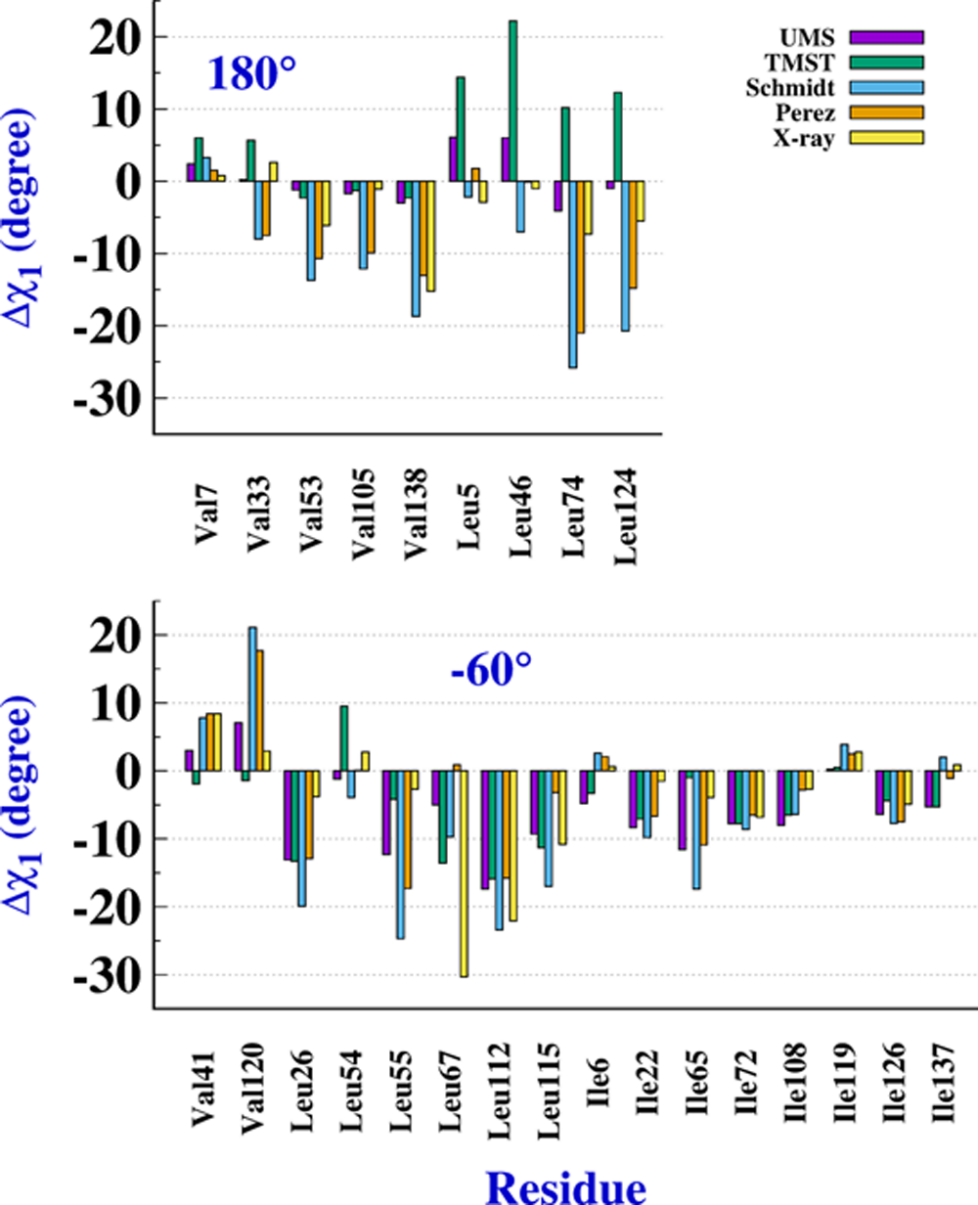
Angular deviation, Δχ_1_, with respect to both staggered angles, 180° (upper plate) and −60° (lower plate) for Val, Leu, and Ile residues.

Figure 5 and Table 1 include results obtained after satisfying two criteria: (a) χ_1_ angles calculated with the UMS model are within the intervals 60 ± 30, 180 ± 30, or −60 ± 30° and (b) a conformer population calculated with the TMST model is greater than 60%, that is, there is a predominant conformer. Results of the remaining four residues that do not meet any of the above criteria will be discussed below. That is, the majority of the side-chain rotamers (25 out of 29) present a dominant χ_1_ conformation in solution. A summary of these results is presented in Figure 5, where the angular deviations of the 25 unimodal residues are displayed. All results are considered in detail in Table 1. Findings about the four exceptional residues are analyzed in subsection “trimodal residues” (see Table 4).

**Table 1:** Optimized Side-Chain Torsion Angle χ_1_ (degree) for 25 (Val, Leu, and Ile) Residues in *D. vulgarisFlavodoxin*^a^

		Unimodal-Static		Trimodal-Static-Staggered				Trimodal-Static-Trigonal						NMR^b^		X-ray^c^
residue		χ_1_	rmsd_*J*,res_^d^	*P*_60_	*P*_180_	*P*_–60_	rmsd_*J*,res_^d^	χ_1_	*P*≈_60_	*P*≈_180_	*P*≈_–60_	rmsd_*J*,res_^d^	*N*_*J*_^e^	Schmidt	Pérez	average
1	Val7	–177.6(+8/+8)	0.80^1^	8	87	5	0.51	–174.0	0	86	14	0.50	7	–176.7 ± 30.3	–178.5 ± 25.1	–179.2 ± 5.7 (13)
2	Val33	–179.8(+8/+8)	0.65^1^	6	82	12	0.32	–174.3	0	80	20	0.29	9	172.0 ± 32.0	172.5 ± 27.8	–177.4 ± 3.6 (13)
3	Val41	–57.0(+8/+8)	0.62^1^	2	13	85	0.41	–61.9	0	16	84	0.41	9	–52.2 ± 32.3	–51.6 ± 24.7	–51.6 ± 7.7 (11)
4	Val53	178.8(+7/+7)	0.54^1^	3	90	8	0.35	177.7	6	90	4	0.35	8	166.3 ± 25.5	169.3 ± 21.7	173.9 ± 3.4 (13)
5	Val105	178.3(+5/+5)	0.26^1^	0	96	4	0.21	178.7	0	98	2	0.20	8	167.9 ± 20.7	170.1 ± 15.8	178.9 ± 6.6 (13)
6	Val120	–52.9(+9/+9)	0.73^1^	1	22	77	0.38	–61.4	0	24	76	0.38	9	–38.9 ± 31.3	–42.3 ± 25.5	–57.1 ± 9.8(10)
7	Val138	177.0(+8/+9)	0.93^1^	2	85	14	0.59	177.7	5	85	10	0.59	7	161.3 ± 23.9	167.0 ± 22.3	164.8 ± 8.6 (13)
8	Leu5	–173.9(+9/+10)	1.30^1^	15	81	4	0.80	–165.6	0	78	22	0.69	7	177.8 ± 27.4	–178.2 ± 19.9	177.1 ± 7.6 (13)
9	Leu26	–73.1(+8/+9)	0.92^1^	20	0	80	0.55	–73.3	6	9	85	0.43	7	–79.9 ± 14.1	–72.9 ± 0.2	–63.8 ± 5.0 (12)
10	Leu46	–174.0(+11/+12)	1.53^2^	18	73	9	0.75	–157.8	0	66	34	0.60	7	173.0 ± 32.8	179.9 ± 27.3	179.0 ± 7.0 (13)
11	Leu54	–61.2(+14/+12)	1.57^1^	23	5	72	0.59	–50.5	33	0	67	0.56	6	–63.9 ± 28.1	–59.9 ± 23.2	–57.2 ± 5.3 (13)
12	Leu55	–72.3(+11/+12)	1.47^1^	23	0	77	0.89	–64.2	19	0	81	0.87	7	–84.7 ± 0.3	–77.3 ± 0.1	–62.7 ± 8.5 (12)
13	Leu67	–65.0(+13/+15)	1.45^1^	14	15	71	0.42	–73.6	4	31	66	0.37	7	–69.7 ± 32.6	–59.1 ± 27.7	–90.3 ± 9.0 (12)
14	Leu74	175.9(+9/+10)	1.24^2^	8	75	18	0.52	–169.8	0	70	30	0.46	8	154.2 ± 13.8	159.0 ± 12.5	172.7 ± 5.3 (12)
15	Leu112	–77.4(+7/+7)	0.79^1^	22	0	78	0.49	–75.9	4	9	86	0.42	6	–83.4 ± 0.3	–75.8 ± 8.4	–82.1 ± 14.5 (13)
16	Leu115	–69.3(+14/+16)	1.56^1^	23	7	71	0.55	–71.3	12	17	71	0.53	7	–77.0 ± 30.5	–63.2 ± 29.2	–70.8 ± 11.5 (13)
17	Leu124	179.0(+10/+10)	1.44^2^	8	77	15	0.74	–167.7	0	71	29	0.67	7	159.3 ± 17.8	165.2 ± 15.6	174.5 ± 3.5 (13)
18	Ile6	–64.8(+8/+8)	0.57^1^	10	0	90	0.40	–63.3	7	0	93	0.38	7	–57.4 ± 22.5	–57.9 ± 14.6	–59.4 ± 5.1 (13)
19	Ile22	–68.3(+6/+6)	0.30^1^	12	0	88	0.34	–67.1	5	0	95	0.18	7	–69.8 ± 20.9	–66.7 ± 15.0	–61.5 ± 8.2 (13)
20	Ile65	–71.6(+11/+11)	0.93^1^	32	0	68	0.18	–61.0	31	0	69	0.18	6	–77.4 ± 35.1	–70.9 ± 33.9	–63.9 ± 3.5 (11)
21	Ile72	–67.8(+4/+4)	0.20^1^	8	0	92	0.38	–67.8	0	0	100	0.12	5	–68.6 ± 16.1	–66.5 ± 0.2	–66.8 ± 3.0 (13)
22	Ile108	–68.0(+7/+7)	0.44^1^	13	0	87	0.38	–66.5	7	0	93	0.28	7	–66.4 ± 22.7	–62.8 ± 16.1	–62.7 ± 3.2 (13)
23	Ile119	–59.8(+7/+7)	0.59^1^	1	0	99	0.46	–59.5	1	0	99	0.46	8	–56.1 ± 18.1	–57.5 ± 0.3	–57.2 ± 6.0 (13)
24	Ile126	–66.4(+8/+9)	0.63^1^	12	0	88	0.49	–64.4	8	0	92	0.45	8	–67.7 ± 18.6	–67.5 ± 11.0	–64.9 ± 6.3 (13)
25	Ile137	–65.3(+6/+6)	0.43^1^	2	0	98	0.43	–65.3	0	0	100	0.32	7	–58.0 ± 0.0	–61.1 ± 0.0	–59.1 ± 3.9 (13)

a Results obtained with the Fourier coefficients calculated at the SOPPA(CCSD)/6-311++G**J level on the α-conformer.

b Results obtained from NMR Karplus parameterization by Schmidt et al.^31^ and Pérez et al.^30^

c Average X-ray results, see the text. Between parentheses, the number of X-ray results is included in the average.

d See eqs 5–7.

e Number of available experimental SSCCs.

As shown in Figure 5, deviations Δχ_1_ from the staggered angles predicted with the UMS and TMST models, two empirical NMR results, and X-ray data are summarized. In the upper plate, we present the results considering an angle of 180° for the staggered conformer as a reference for nine residues (five Val and four Leu). In general, with some exceptions, the differences go in the same direction, and the UMS model predicts small values and the same sign as the X-ray values. The differences found in the values of the TMST model for the four Leu residues are also notable. On the bottom plate of Figure 5, we present the equivalent results to the previous ones with respect to a staggered angle of −60°. 15 residues belong to this group (two Val, five Leu, and seven Ile). For this set of Δχ_1_, as in the previous set, the UMS model predicts the same sign as the X-ray values, showing greater differences in the Leu residues and outstanding the good agreement of the Ile residues.

The full description of results for the 25 residues of Figure 5 is given in Table 1, where each column is explained below. Column #1 in Table 1 defines the residues. Columns #2 and #3 show results obtained using the UMS model; χ_1_ values correspond to the minima in the curves of rmsd_*J*,res_. The representation of rmsd_*J*,res_ versus χ_1_ for all residues is shown in Figure S1 (Supporting Information). They generally exhibit two minima due to the intrinsic degeneracy of the Karplus equation.^32^ For the majority of residues, the χ_1_ angles, as shown in column #2 of Table 1, correspond to the absolute minima, that is, the first minimum, indicated by a superindex (^1^) together with the minimum rmsd_*J*,res_ in column #3. For three residues, Leu46, Leu74, and Leu124, the considered χ_1_ angle corresponds to a second minimum (superindex ^2^). Moreover, in column #3, we present an estimation of the χ_1_ uncertainty based on the shape of rmsd_*J*,res_ curve. These uncertainties are obtained considering the angles around the minimum included in a rmsd_*J*,res_ corresponding to the minima plus 0.2 Hz, giving an idea of how flat or steep the rmsd_*J*,res_ curve is.

Columns #4 to #7 in Table 1 show the results derived from the TMSS model. Columns #4 to #6 present the populations (%) corresponding to the staggered rotamers. The χ_1_ angle used to compare with the other two models corresponds to that of maximum population, larger than 60%. It should be noted that this model does not predict any residue with a dominant conformation around 60°. The maximum population for a χ_1_ = 60° rotamer is 32% for the Ile65 residue. The rmsd_*J*,res_ values, corresponding to the minimum and considering the three staggered rotamers, are shown in column #7.

Columns #8 to #12 show results achieved using the TMST model. The χ_1_ angle, as shown in column #8, corresponds to the rotamer with the highest population, the other two χ_1_ angles are χ_1_ + 120 and χ_1_ – 120°. The rmsd_*J*,res_ values for both trimodal models are similar, and obviously both are smaller than those of the UMS model where only one parameter is optimized.

Column #13, labelled *N*_*J*_, shows the number of available experimental SSCCs. Next columns present empirical results of Schmidt et al.^31^ and Pérez et al.^30^ and those obtained from X-ray studies.^36^ χ_1_ average X-ray angles, as shown in the last column of Table 1, are calculated from more than 10 X-ray values extracted from the PDB^36^ and from those collected by Schmidt.^31^ In fact, only one average value is obtained with 10 individual X-ray angles, two from 11, four from 12, and the remaining ones, 18 average values, were obtained from 13 individual values. This means that the agreement between the different X-ray studies is very satisfactory for all residues. The rmsd values are smaller than 10° except for two residues whose rmsd values amount 14.5° (Leu112) and 11.5° (Leu115). These residues and Leu67 present the highest deviations (22.1, 10.8, and 30.3°, respectively) with respect to the staggered (−60°) conformer. It should be noted that deviations with respect to the TMST model (6.6, 0.5 and 16.7°, respectively) are smaller.

Table 2 summarizes the deviations of the different methods or models. Dihedral angles, χ_1_, obtained in this work are in good agreement with those previously obtained from X-ray structures^31^ and from empirical NMR Karplus equations.^30,31^ χ_1_ angles obtained with the UMS model compared with X-ray and with both empirical NMR results present rmsd_χ1_ values of 7.6, 9.8, and 6.9° (Table 2). Deviations for χ_1_ angles of the three models for Val, Leu, and Ile residues with respect to X-ray values are considered in Figure S2 (Supporting Information). The UMS model yields the best agreement when compared with the X-ray angles.

**Table 2:** rmsd_χ_1__ Values (degree), eq 9, between the Indicated Results^a^

	rmsd_χ1_					
	TMSS	TMST	Schmidt	Pérez	X-ray	rmsd_*J*,tot_^α^
UMS	7.3^17^	7.0^16^	9.8^22^	6.9^17^	7.6^25^	0.96
TMSS		9.2^22^	14.1^26^	10.0^21^	9.2^30^	0.68
TMST			15.4^36^	12.6^31^	9.7^23^	0.61
Schmidt				5.5^14^	10.6^22^	
Pérez					9.3^31^	

a α-conformer is considered. Superindex corresponds to the maximum deviation. The last column shows the rmsd_*J*,tot_ values (Hz), eq 8, for the results of this work.

It is remarkable to note that χ_1_ angles obtained in this work using NMR experimental SSCCs and theoretical Karplus coefficients are more similar to those obtained by X-ray structures than those obtained with the same set of experimental couplings and empirical Karplus coefficients.^30,31^ It is also noteworthy that TMSS and TMST models present larger χ_1_ deviations. Despite these models yield a good agreement when compared to experimental and calculated SSCCs, see rmsd_*J*,res_ in Table 1 and rmsd_*J*,tot_ values in Table 2. However, it should be noted that these χ_1_ deviations are similar or slightly smaller than those of Schmidt and Pérez (see rmsd_χ_1__ values in Table 2).

**Figure 6:**
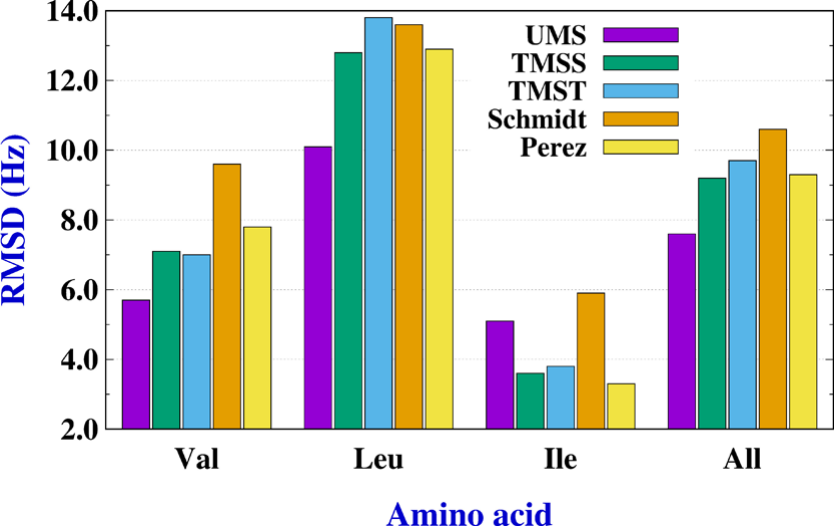
values (rmsd between the χ_1_ angles calculated with the indicated method and those obtained as average X-ray) for Val, Leu, Ile, and all residues.

Figure 6 shows the χ_1_ deviations against the X-ray average, that is, , derived from the different methods and for the three AAs. Numerical values can be found in Table S10. The largest deviations, irrespectively of used method, correspond to Leu residues, while the lowest deviations are those of Ile residues. In this last case, the rmsd values of Pérez et al.^30^ are smaller than those calculated with the other methods. For Val, Leu, and the whole set, the best results are those of the UMS method. However, for Ile, the best results are those of Pérez, TMSS, and TMST.

In Table 3, we summarize the final results of the popular B3LYP and a selection of the best functionals to calculate SSCCs^23^ (B972, S55VWN5, and wB98xD) comparing them with WF SOPPA(CCSD) results. We show two statistical parameters: the  obtained by comparison with the X-ray χ_1_ values and the rmsd_*J*,tot_. The best results are clearly those of the WF method. Nevertheless, some functionals perform accurately, and the differences with WF results are small. To simplify, we focus our attention on the results of the α-conformer. When appropriate, we will indicate some of the highlights of the beta conformer. Within the UMS model, the  values amount 7.6°, while the DFT values are close to 7.9 or 8.0° except for B3LYP that amounts 9.5°. For this statistical parameter, results for the β-conformer are slightly worse. rmsd_*J*,tot_ values are 0.96 Hz for SOPPA(CCSD) and between 1.04 and 1.10 Hz for B972, S55VWN5, and wB98xD, while the one corresponding to B3LYP increases to 1.42 Hz. In contrast to  values, the rmsd_*J*,tot_ values for the β-conformer are better than those of the α-conformer. This behavior is reproduced also using TMST and TMSS models.

**Table 3:** Summary of Results Obtained by the Indicated WF and DFT Methods^a^

		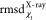			rmsd_*J*,tot_		
		UMS	TMSS	TMST	UMS	TMSS	TMST
SOPPA(CCSD)	α	7.6^25^ (25)	7.0^22^ (24)	9.7^23^ (25)	0.96 (181)	0.68 (181)	0.61 (181)
	β	7.7^25^ (25)	7.0^22^ (24)	9.4^19^ (25)	0.84 (181)	0.54 (181)	0.45 (181)
B3LYP	α	9.5^19^ (24)	7.1^22^ (23)	9.2^20^ (19)	1.42 (175)	1.01 (175)	0.90 (175)
	β	10.3^19^ (24)	7.1^22^ (23)	14.7^30^ (21)	1.22 (175)	0.80 (175)	0.65 (175)
B972	α	8.0^28^ (25)	7.0^22^ (24)	11.1^28^ (25)	1.07 (181)	0.76 (181)	0.69 (181)
	β	8.1^27^ (25)	7.0^22^ (24)	10.3^21^ (25)	0.91 (181)	0.60 (181)	0.51 (181)
S55VWN5	α	8.0^25^ (25)	7.0^22^ (24)	11.6^30^ (25)	1.10 (181)	0.77 (181)	0.70 (181)
	β	8.3^25^ (25)	7.0^22^ (24)	10.8^22^ (25)	0.92 (181)	0.59 (181)	0.50 (181)
wB98xD	α	7.9^28^ (25)	7.0^22^ (24)	10.8^28^ (25)	1.04 (181)	0.76 (181)	0.70 (181)
	β	8.0^27^ (25)	7.0^22^ (24)	10.0^21^ (25)	0.88 (181)	0.61 (181)	0.52 (181)

a (degree), eq 9, between the X-ray angles and those calculated with the indicated model and rmsd_*J*,tot_ (Hz), eq 8, between the experimental SSCCs and those calculated with the indicated model. Results from WF (6-311++G** basis set) and DFT methods (aug-cc-pVTZ-J basis set) are shown. For comparison,  between X-ray average results and those of Schmidt and Pérez are, respectively, 10.6^+22^ (22) and 7.1^+15^ (25) and those between Schmidt and Pérez, both obtained by NMR, are 5.5^+14^ (25).

The TMSS model presents similar results for all methods with an  around 7.0°. It seems to give better results than the UMS and the TMST models, although it should be considered that deviations larger than 30° were removed. In the TMSS model, at least the Leu67 value that deviates 30.3° has been removed. If this value were included, the  would be higher. rmsd_*J*,tot_ values for the trimodal models are clearly better than those of the unimodal model. However, we must also consider that the number of optimized parameters increases.

Surprisingly, the TMST model predicts χ_1_ values worse than those of the UMS model. Ab initio result for  is 9.7° (9.4 for β-conformer), and the DFT results are worse between 10.8 and 11.6° for B972, S55VWN5, and wB98xD functionals.

### Trimodal Residues

As indicated above, results presented in Table 1 include all residues that meet certain criteria (χ_1_ angle close staggered values and conformer population larger than 60%). Only four residues of a total of 29 (14%) do not meet one or both criteria. These residues are shown in Table 4, and they should be analyzed cautiously. The column definition is similar to that previously described for Table 1. For them, a reliable interpretation can be obtained considering either the small number of experimental available SSCCs and/or the possibility of two or three conformers around χ_1_. For this reason, we call them trimodal residues. Thus, the four residues Val88, Val144, Leu78, and Ile148 do not meet the population criteria, that is, one conformer with more than 60% of population. Two of these residues (Val88 and Ile148) show two dominant conformers (the third and smallest conformational population was predicted as 13 and 8%, respectively). For the other two residues, three conformers with no negligible population should be considered (the smallest conformer population is now 18 and 26%, respectively, within the TMSS model), see Table 4.

**Table 4:** Optimized Side-Chain Torsion Angle χ_1_ (degree) for Four Trimodal Residues in *D. vulgarisFlavodoxin*^a^

		Unimodal-Static		Trimodal-Static-Staggered				Trimodal-Static-Trigonal						NMR^b^		X-ray^c^
residue		χ_1_	rmsd_*J*,res_^d^	*P*_60_	*P*_180_	*P*_–60_	rmsd_*J*,res_^d^	χ_1_	*P*_≈60_	*P*_≈180_	*P*≈−_60_	rmsd_*J*,res_^d^	*N*_*J*_^e^	Schmidt	Pérez	average
1	Val88	164.6(+14/+13)	1.86^1^	13	42	46	0.46	172.8	13	47	40	0.46	5	–10.8 ± 25.7	133.7 ± 25.8	173.4 ± 4.3(4)
		–42.4(+18/+17)	1.91^2^													–57.1 ± 8.3 (8)
2	Val144	166.7(+21/+20)	1.61^1^	18	43	39	0.45	163.5	22	49	30	0.42	9	131.9 ± 20.4	133.5 ± 30.7	164.7 ± 3.0 (7)
		–35.9(+19/+18)	1.61^2^													–45.6 ± 14.3 (6)
3	Leu78	100.2(+20/+24)	2.50^2^	33	26	41	0.78	–90.0	26	31	43	0.60	9	–111.6 ± 30.1	–109.6 ± 44.1	–140.7 ± 11.8 (9)
		–100.9(+24/+26)	2.30^1^													–85.4 ± 5.6 (4)
4	Ile148	72.4(+13/+13)	1.46^1^	53	8	39	0.45	–72.6	41	8	51	0.42	7	104.3 ± 27.9	99.6 ± 28.8	56.8 ± 10.2 (8)
		–84.5(+16/+15)	1.55^2^													–67.7 ± 6.1 (4)

a Results obtained with the Fourier coefficients calculated at the SOPPA(CCSD)/6-311++G**J level on the α-conformer.

b Results obtained from an NMR Karplus parameterization by Schmidt et al.^31^ and Pérez et al.^30^

c Average X-ray results, see the text. Between parentheses, the number of X-ray results is included in the average.

d See eqs 5–7.

e Number of available experimental SSCCs.

For Val88, we predict two conformers with χ_1_ around 180 and −60°, and with populations of 42 and 46%, respectively, when using the UMS model, and 47 and 40%, respectively, when using the TMST model. It should also be noted that for Val88 only five experimental SSCCs are available, those involving proton, which make an accurate interpretation more difficult. For Val88, the UMS model predicts two unimodal (100% populated) χ_1_ angles of 165 or −42°, while the TMST model predicts 173° (47%) and −67° (40%). These figures are in good agreement with the two available X-ray averages of 173 and −57°.

Ile148 shows a similar behavior, but now the two main conformers around 60 and −60° should be considered. The UMS model found two minima χ_1_ angles at 72 and −85°. The TMSS model predicts populations of 53% (for 60° conformer) and 39% (for −60°). Using the TMSS, the χ_1_ angles (population) are 47° (41%) and −73° (51%). X-ray values are 57 and −68° averaging eight and four PDB entries, respectively.

The UMS model predicts χ_1_ angles of 167 or −36° for Val144. Moreover, the TMSS model predicts populations of 18% (60°), 43% (180°), and 39% (−60°), and the TMST model yields the following angles (populations): 44° (22%), 164° (49%), and −76° (30%) that are in reasonable agreement with the X-ray angles (165 and −46°). The angle −76° (close to 60°), corresponding to the lowest population, is not found in the X-ray results.

Leu78 seems to be more complicated since a single conformer model yields two possible minima with quite anomalous χ_1_ angles (−100.9 and 100.2°). None of these angles fulfill the angle criterium considered previously. It should be noted that both previous NMR studies^30,31^ predict χ_1_ angles of −111.6 and −109.4° with large deviations of 30 and 44°, respectively. In the TMSS model, we predict three possible conformers 60° (33%), 180° (26%), and −60° (41%), and the TMSS model also yields three conformers 30° (26%), 150° (31%), and −90° (43%). The X-ray χ_1_ angle is −141° when nine PDB entries are averaged, and −85.4° when the four remaining PDB entries are considered. The rmsd_*J*,RE_ values, see eqs 5 and 6, are reduced to one-third when using the trimodal model with respect to the unimodal model.

## Conclusions

A computational procedure for obtaining conformational side-chain information of dipeptides using SSCCs combined with Karplus equations and quantum chemistry methods has been developed.

Initially, a detailed analysis about the different factors that affects the calculated SSCCs is presented. The 6-31++G**-J basis set shows a similar quality to the larger aug-cc-pVTZ-J. Both basis sets were specifically developed to calculate SSCCs. The backbone and the side-chain χ_2_ conformation also affect the resulting Fourier coefficients, and these effects could be considered in a detailed study. Differences are found when Fourier coefficients are compared with empirical ones. In spite of these differences and factors, theoretical Fourier coefficients predict χ_1_ angles better than empirical ones. It should be noted that the above factors affect both theoretical and empirical parameterizations. The performance of a selected set of functionals is compared with that of expensive WF methods. The functional S55VWN5,^28^ specifically developed for SSCCs, presents the best results. Nevertheless, standard functionals B972 and wB97XD also show good performance. Any of these three functionals could be used for faster and more cost-effective studies.

A combination of experimental ^3^*J*_*XY*_^exp^ with theoretical Karplus equations is used to determine the χ_1_ side-chain dihedral angles establishing three different models. These models (UMS, TMSS, and TMST) have been applied to study relationships between vicinal SSCCs and torsion angles for Val, Leu, and Ile residues and validated with experimental NMR and X-ray data. An UMS model considers a single conformer and minimizes the rmsd, eq 5, for each residue to predict the χ_1_ angle. The TMSS model contemplates three staggered conformers for χ_1_ (60, 180, and −60°) and determines their populations (two parameters). The TMST model considers trigonal symmetry and computes three parameters (two populations and one χ_1_ angle) by least squares fitting. Side-chain torsion angle χ_1_ has been optimized for 29 Val, Leu, and Ile residues achieving successful results for 25 of them, with an excellent agreement with X-ray angles. The four discordant residues (Val88, Val144, Leu78, and Ile148) have been thoroughly studied, showing that they do not have a unique conformation and that the population of conformers plays an important role. These four residues do not meet the population criterium, that is, none of the conformer contributes with more than 60% of population. It is relevant that these four trimodal residues present a good agreement with the X-ray averages. For those four residues, X-ray data present at least two conformers, showing a trimodal character. We conclude that most of these so-called trimodal residues present multiple conformations and that the methods developed in this work are helpful for detecting these special residues.

We consider that the procedure and the Karplus equations developed in this work used in *D. vulgarisFlavodoxin* residues can be utilized for Val, Leu, and Ile residues of any other protein as long as the experimental coupling constants are available.

## Data and Software Availability

A new computational procedure is described in this work. The computational strategy and different models proposed here using the workflow allow us to get the final results. The different conformers necessary to carry out this study, according to the workflow, are obtained from standard optimized geometries.

Software and standard packages are well known. They are owned by their respective developers and copyright holders. We have referenced and provided the appropriate links.

All data and results can be easily reproduced following the corresponding instructions and using the standard computational packages indicated in this work. Optimized geometries are presented in http://rmn5.qfa.uam.es/geo.

## Supporting Information Available

Torsion angles χ_1_ obtained from the X-ray PDB entries; deviations from tetrahedral angle; Fourier coefficients calculated at SOPPA(CCSD)/6-31++G**; Fourier coefficients calculated at DFT level for Val, Leu, and Ile; Fourier coefficient collection; statistical parameter for different factors about calculated SSCCs; representation of rmsd_*J*,RE_ values versus χ_1_; and deviation of χ_1_ calculated and those derived from X-ray (PDF)
