# Supplementary material for: Toward a Computational NMR Procedure for Modeling Dipeptide Side-Chain Conformation
Source: J Chem Inf Model. 2021 Nov 11;61(12):6012–23. doi: 10.1021/acs.jcim.1c00773 (PMC8715507; doi:10.1021/acs.jcim.1c00773)
Supplement: Supplementary file 1 — ci1c00773_si_001.pdf [file ci1c00773_si_001.pdf]

Toward a Computational NMR Procedure for Modeling Dipeptides Side Chain Conformation (Supporting Information)

Jesús San Fabián, Ignacio Ema, Salama Omar and Jose Manuel García de la Vega

Table S1: Torsion angles χ_1 (deg) for Val, Leu and Ile residues in *Desulfovibrio vulgaris* flavodoxin obtained from the indicated X-ray PDB[a] entries[b] except the Walsh data[c].

Residue	Walsh	1BU5a	1BU5b	1J8Q	2FX2	3FX2	4FX2	5FX2	1F4P	1J9E	5YOE	5YOG	5YOB	average	n[d]
ILE006	-63.8	-62.7	-59.6	-54.3	-69.5	-62.8	-55.1	-52.0	-64.4	-53.5	-55.9	-59.7	-58.5	-59.4± 5.1	13
ILE022	-67.6	-66.4	-60.5	-61.4	-63.0	-53.8	-61.1	-37.6	-68.7	-60.7	-65.4	-67.6	-65.3	-61.5± 8.2	13
ILE065	-71.7	114.8	142.5	-65.2	-66.1	-59.9	-62.2	-66.0	-62.9	-63.5	-59.8	-65.3	-60.1	-63.9± 3.5	11
ILE072	-59.8	-66.8	-67.0	-70.8	-64.7	-67.6	-63.2	-65.1	-67.7	-68.8	-69.1	-68.4	-70.0	-66.8± 3.0	13
ILE108	-66.8	-65.4	-63.3	-62.7	-63.6	-61.0	-53.8	-60.7	-63.6	-63.1	-61.6	-66.1	-63.2	-62.7± 3.2	13
ILE119	-63.0	-54.7	-53.3	-48.6	-67.6	-49.4	-60.2	-63.6	-56.0	-49.3	-59.1	-60.8	-57.8	-57.2± 6.0	13
ILE126	-60.9	-70.4	-74.5	-62.7	-66.0	-72.4	-59.4	-74.2	-66.2	-63.5	-56.5	-60.0	-57.3	-64.9± 6.3	13
ILE137	-66.3	-59.3	-62.5	-58.4	-57.9	-54.1	-50.6	-58.5	-59.3	-58.6	-58.8	-60.9	-62.8	-59.1± 3.9	13
ILE148	62.2	91.0	68.2	61.2	57.0	61.1	52.3	34.1	-67.6	57.5	-66.5	-69.5	-67.2	56.8± 10.2	8
LEU005	170.9	173.0	171.4	175.5	-168.8	177.8	178.1	-164.9	172.4	177.8	171.9	174.4	172.9	177.1± 7.6	13
LEU026	-96.4	-57.5	-72.4	-65.5	-66.4	-72.0	-60.4	-67.9	-61.4	-61.2	-58.4	-61.0	-61.3	-63.8± 5.0	12
LEU046	-178.2	-171.6	-179.4	-176.8	179.2	172.7	165.2	166.2	-174.3	179.7	178.6	-176.9	-176.8	179.0± 7.0	13
LEU052	179.1	172.6	179.9	172.9	176.7	-163.7	-174.8	-174.8	174.8	175.4	172.1	172.0	173.6	178.1± 7.1	13
LEU054	-65.8	-55.2	-60.1	-51.1	-68.9	-52.8	-53.8	-55.0	-60.2	-54.2	-55.9	-57.3	-53.3	-57.2± 5.3	13
LEU055	-64.2	-58.5	-59.3	-58.4	-99.5	-67.6	-61.5	-88.2	-59.3	-59.4	-57.5	-58.9	-60.7	-62.7± 8.5	12
LEU067	-97.3	-92.7	-97.1	-90.4	-93.0	-102.8	-94.4	-35.7	-66.7	-88.3	-87.7	-85.2	-87.5	-90.3± 9.0	12
LEU074	174.0	168.5	170.1	174.0	174.1	170.4	170.5	160.9	177.6	173.1	—	178.5	-179.2	172.7± 5.3	12
LEU078	-87.2	-134.7	-136.6	-91.2	-125.5	-76.1	-131.9	-136.2	-134.7	-87.1	-159.9	-151.0	-155.7	-140.7± 11.8	9
LEU112	-73.4	-111.9	-103.5	-74.6	-104.7	-79.8	-81.4	-66.0	-72.6	-73.3	-75.8	-75.3	-77.2	-82.1± 14.5	13
LEU115	-74.8	-54.2	-54.4	-69.5	-84.3	-81.3	-67.3	-95.8	-64.9	-73.0	-64.5	-69.8	-67.3	-70.8± 11.5	13
LEU124	177.8	174.6	166.3	174.6	175.9	173.7	179.2	169.8	173.7	178.4	173.6	173.2	177.1	174.5± 3.5	13
VAL007	-171.7	-177.2	-173.1	-178.9	-172.5	-171.8	176.4	169.1	178.1	179.0	176.4	178.3	178.3	-179.2± 5.7	13
VAL033	-179.7	179.7	-179.6	-174.8	175.5	179.7	-174.3	-169.8	-176.8	-175.9	-175.5	-178.2	-176.6	-177.4± 3.6	13
VAL041	-53.2	-54.7	-54.3	-58.8	-51.6	-29.9	156.0	107.3	-54.3	-58.1	-52.1	-48.6	-51.6	-51.6± 7.7	11
VAL053	176.0	178.3	176.2	175.1	177.3	167.5	170.3	168.2	173.7	176.8	173.0	172.6	175.6	173.9± 3.4	13
VAL088	-179.6	-50.9	-56.6	-50.8	-59.0	-56.5	-49.8	-75.7	-172.0	-58.0	-172.6	-4.7	-169.6	-57.1± 8.3	8
VAL105	-179.2	-175.9	-179.0	175.6	-170.2	-172.1	176.4	-174.5	172.5	179.7	170.6	169.8	172.6	178.9± 6.6	13
VAL120	-53.6	132.9	143.5	-63.1	-53.0	-53.7	-59.5	-33.1	-62.5	-59.7	-0.2	-65.5	-67.1	-57.1± 9.8	10
VAL138	168.4	160.1	173.8	159.5	163.7	166.0	146.0	150.6	170.8	170.6	170.7	169.7	171.9	164.8± 8.6	13
VAL144	162.6	-28.2	-32.6	-58.7	-38.1	-54.5	159.2	168.0	163.9	-61.3	166.2	166.8	166.1	164.7± 3.0	7

[a] H. M. Berman, J. Westbrook, Z. Feng, G. Gilliland, T. N. Bhat, H. Weissig, I. N. Shindyalov, and P. E. Bourne, "The Protein Data Bank", *Nucleic Acids Res.*, **28**, 235-242, (2000).

[b] 1BU5a and 1BU5b M.A. Walsh, *et al. Eur. J. Biochem.*, **258**, 362-371 (1998); 1J8Q and 1J9E R Artali *et al. Acta Crystallogr.* Sect. D, **58**, 1787-1792 (2002); 2FX2, 3FX2, 4FX2 and 5FX2 Watt W *et al. J. Mol. Biol.* 1991, **218**, 195-208; 1F4P R.A. Reynolds, *et al. Acta Crystallogr.* Sect. D **57**, 527-535 (2001); 5YOE, 5YOG, 5YOB M. Pu, *et al. Protein Cell* **9**, 659-663 (2018).

[c] J. M. Schmidt, J. Biomol NMR 37, 287-301 (2007).

[d] Data considered in the average. Data neglected (deviations larger than 30 deg) are underlined.

Table S2: Average dihedral angle deviations against the tetrahedral angles, $\theta^{calc} - (\chi_1 + \Delta\theta^{tetrah})$, for the dihedral angles involved in the $C^\alpha - C^\beta$ bond. Minimum and maximum (Min/Max) deviations are also shown. Reference dihedral angle χ_1 is $N' - C^\alpha - C^\beta - C^{\gamma_1}$.

	$H^\alpha - H^\beta$	$H^\alpha - C^{\gamma_1}$	$H^\alpha - C^{\gamma_2}$	$C' - H^\beta$	$C' - C^{\gamma_1}$	$C' - C^{\gamma_2}$	$N' - H^\beta$	$N' - C^{\gamma_2}$
**Val α-helix**								
Average	2.1	-0.8	5.1	-4.6	-7.5	-1.7	2.9	5.9
Min/Max	-3.2/6.7	-3.3/1.3	0.7/9.9	-10.1/-0.4	-11.5/-4.9	-6.1/3.6	0.0/5.6	2.6/8.6
$\Delta\theta^{proposed}$	2.1	119.2	-114.9	115.4	-127.5	-1.7	-117.1	125.9
**Val β-sheet**								
Average	-0.7	-3.5	2.1	1.8	-1.1	4.6	2.8	5.6
Min/Max	-5.0/2.7	-5.7/-1.7	-1.5/6.6	-2.8/5.5	-4.2/1.8	0.4/ 9.4	0.7/4.4	2.1/8.2
$\Delta\theta^{proposed}$	-0.7	116.5	-117.9	121.8	-121.1	4.6	-117.2	125.6
	$H^\alpha H^{\beta_2}$	$H^\alpha - H^{\beta_3}$	$H^\alpha - C^{\gamma_1}$	$C' - H^{\beta_2}$	$C' - H^{\beta_3}$	$C' - C^{\gamma_1}$	$N' - H^{\beta_2} -$	$N' - H^{\beta_3}$
**Leu α-helix**								
Average	2.5	-4.0	-0.7	-4.0	-10.4	-7.2	3.2	-3.2
Min/Max	-3.1/6.4	-11.3/1.0	-5.4/1.9	-11.1/2.1	-19.4/-3.5	-13.4/-3.1	-0.6/5.7	-6.8/-0.2
$\Delta\theta^{proposed}$	-117.5	-4.0	119.3	-4.0	109.6	-127.2	123.2	-123.2
**Leu β-sheet**								
Average	-0.1	-6.4	-3.3	2.7	-3.6	-0.5	3.2	-3.1
Min/Max	-4.7/4.1	-12.0/-1.8	-7.4/-0.7	-3.5/8.9	-10.6/2.4	-6.0/3.0	-0.2/6.0	-6.1/-0.4
$\Delta\theta^{proposed}$	-120.1	-6.4	116.7	2.7	116.4	-120.5	123.2	-123.1
	$H^\alpha H^\beta$	$H^\alpha - C^{\gamma_1}$	$H^\alpha - C^{\gamma_2}$	$C' - H^\beta$	$C' - C^{\gamma_1}$	$C' - C^{\gamma_2}$	$N' - H^\beta$	$N' - C^{\gamma_2}$
**Ile α-helix**								
Average	-4.7	-1.1	-8.4	-11.5	-7.9	-15.2	-3.6	-7.4
Min/Max	-10.9/-0.1	-4.1/1.4	-17.4/-2.1	-19.8/-5.2	-13.0/-3.7	-26.3/-7.2	-7.7/-0.2	-13.3/-3.0
$\Delta\theta^{proposed}$	-124.7	118.9	-8.4	-11.5	-127.9	104.8	116.4	-127.4
**Ile β-sheet**								
Average	-7.0	-3.6	-10.7	-4.8	-1.3	-8.4	-3.5	-7.1
Min/Max	-12.9/-2.3	-6.2/-1.0	-17.9/-4.5	-11.1/1.6	-5.6/2.5	-17.1/-0.7	-7.4/0.2	-12.0/-3.2
$\Delta\theta^{proposed}$	-127.0	116.4	-10.7	-4.8	-121.3	111.6	116.5	-127.1

Table S3: Fourier coefficients[a] for the indicated SSCCs of calculated at the SOPPA(CCSD)/6-311++G**-J for the α-helix and β-sheet backbone conformations.

	α-helix						β-sheet					
SSCC	C_0	C_1	C_2	C_3	S_1	S_2	C_0	C_1	C_2	C_3	S_1	S_2
**Valine**												
$^3J_{H^\alpha H^\beta}$	6.19	0.93	5.21	-0.11	-0.12	0.96	5.76	0.63	4.95	-0.18	0.08	0.19
$^3J_{H^\alpha C^{\gamma_1}}$	3.73	0.64	3.46	0.11	0.18	0.16	3.46	0.50	3.11	0.03	0.11	-0.15
$^3J_{H^\alpha C^{\gamma_2}}$	3.75	0.46	3.44	-0.02	-0.16	1.12	3.47	0.37	3.25	-0.03	-0.13	0.66
$^3J_{C'H^\beta}$	3.65	-1.04	3.39	-0.01	0.31	-1.20	3.50	-1.32	3.34	-0.15	0.26	-0.64
$^3J_{C'C^{\gamma_1}}$	1.65	-0.79	1.29	-0.04	0.50	-0.90	1.59	-0.95	1.45	-0.18	0.39	-0.60
$^3J_{C'C^{\gamma_2}}$	1.69	-0.69	1.57	0.10	0.17	-0.47	1.60	-0.68	1.50	0.10	0.23	-0.20
$^3J_{N'H^\beta}$	-2.47	-0.32	-2.42	-0.14	0.10	-0.09	-2.15	0.27	-2.06	-0.04	-0.03	-0.13
$^3J_{N'C^{\gamma_1}}$	-1.17	-0.26	-1.21	-0.13	-0.08	0.11	-1.13	-0.02	-1.17	-0.10	-0.07	0.04
$^3J_{N'C^{\gamma_2}}$	-1.17	-0.30	-1.14	-0.10	0.20	-0.24	-1.15	-0.02	-1.12	-0.06	0.22	-0.31
**Leucine**												
$^3J_{H^\alpha H^{\beta_2}}$	6.73	0.35	5.84	-0.19	-0.13	1.71	6.26	0.20	5.68	-0.19	0.05	0.73
$^3J_{H^\alpha H^{\beta_3}}$	6.66	0.43	5.73	-0.24	0.04	0.65	6.23	0.28	5.35	-0.22	0.17	-0.17
$^3J_{H^\alpha C^\gamma}$	3.63	-0.62	3.56	-0.10	-0.06	0.62	3.34	-0.57	3.23	-0.05	-0.02	0.23
$^3J_{C'H^{\beta_2}}$	3.96	-1.04	3.66	-0.04	0.17	-1.07	3.79	-1.25	3.58	-0.03	0.13	-0.39
$^3J_{C'H^{\beta_3}}$	3.86	-1.20	3.36	-0.32	0.44	-1.65	3.79	-1.52	3.54	-0.40	0.25	-0.96
$^3J_{C'C^\gamma}$	1.59	-0.79	1.43	-0.10	0.22	-0.59	1.48	-0.95	1.42	-0.10	0.17	-0.34
$^3J_{N'H^{\beta_2}}$	-2.98	0.16	-2.69	-0.04	0.32	-0.49	-2.55	0.63	-2.33	0.05	0.15	-0.52
$^3J_{N'H^{\beta_3}}$	-2.96	0.21	-2.90	0.02	-0.20	0.25	-2.58	0.67	-2.50	0.12	-0.19	0.07
$^3J_{N'C^\gamma}$	-0.96	0.34	-1.04	-0.02	0.07	-0.04	-0.99	0.43	-1.07	0.01	0.08	-0.08
**Isoleucine**												
$^3J_{H^\alpha H^\beta}$	6.36	1.00	5.54	0.07	-0.14	1.01	5.93	0.66	5.20	-0.09	0.03	0.21
$^3J_{H^\alpha C^{\gamma_1}}$	3.65	0.25	3.30	0.08	-0.26	1.12	3.36	0.14	3.11	0.03	-0.15	0.73
$^3J_{H^\alpha C^{\gamma_2}}$	3.80	0.71	3.53	0.14	0.12	0.09	3.53	0.60	3.09	0.07	-0.02	-0.21
$^3J_{C'H^\beta}$	3.71	-0.98	3.40	-0.12	0.41	-1.27	3.57	-1.34	3.42	-0.25	0.29	-0.66
$^3J_{C'C^{\gamma_1}}$	1.61	-0.70	1.51	0.08	0.20	-0.38	1.48	-0.70	1.39	0.02	0.22	-0.12
$^3J_{C'C^{\gamma_2}}$	1.61	-0.79	1.38	-0.23	0.60	-1.07	1.56	-0.96	1.54	-0.29	0.40	-0.72
$^3J_{N'H^\beta}$	-2.56	-0.22	-2.50	-0.11	0.04	-0.13	-2.22	0.38	-2.13	-0.03	0.01	-0.18
$^3J_{N'C^{\gamma_1}}$	-1.02	-0.09	-0.97	-0.11	0.20	-0.27	-1.02	0.09	-1.01	-0.09	0.21	-0.31
$^3J_{N'C^{\gamma_2}}$	-1.22	-0.27	-1.24	-0.06	-0.10	0.11	-1.17	-0.02	-1.20	-0.06	-0.09	0.01

[a] For Leu and Ile, the coefficients correspond to the average between those of the three staggered χ_2 conformers.

Table S4: Fourier coeficients for Val calculated using the indicated method.

	α-conformer						β-conformer					
Method	C_0	C_1	C_2	C_3	S_1	S_2	C_0	C_1	C_2	C_3	S_1	S_2
$^3J_{H^\alpha H^{\beta 3}}$												
SOPPA(CCSD)/6-311++Gee-J	6.19	0.93	5.21	-0.11	-0.12	0.96	5.76	0.63	4.95	-0.18	0.08	0.19
B3LYP/6-311++Gee-J	6.76	1.10	5.80	-0.24	-0.08	1.14	6.33	0.79	5.55	-0.26	0.06	0.24
B3LYP/aug-cc-pVTZ-J	6.80	0.86	5.78	-0.25	-0.07	1.15	6.35	0.55	5.54	-0.26	0.06	0.25
B972/aug-cc-pVTZ-J	6.01	0.75	5.13	-0.24	-0.07	1.02	5.63	0.50	4.92	-0.25	0.05	0.22
B97D/aug-cc-pVTZ-J	5.66	0.80	4.75	-0.25	-0.05	0.98	5.34	0.58	4.57	-0.23	0.05	0.24
S55VWN5/aug-cc-pVTZ-J	6.25	0.70	5.19	-0.18	-0.08	1.02	5.80	0.41	4.94	-0.22	0.07	0.18
wB97X/aug-cc-pVTZ-J	5.94	0.81	5.01	-0.18	-0.07	0.93	5.56	0.55	4.78	-0.22	0.05	0.14
wB97XD/aug-cc-pVTZ-J	6.04	0.82	5.10	-0.20	-0.07	0.97	5.66	0.55	4.88	-0.23	0.06	0.17
$^3J_{H^\alpha C^{\gamma 1}}$												
SOPPA(CCSD)/6-311++Gee-J	3.73	0.64	3.46	0.11	0.18	0.16	3.46	0.50	3.11	0.03	0.11	-0.15
B3LYP/6-311++Gee-J	4.08	0.90	3.82	0.07	0.19	0.14	3.80	0.74	3.46	0.01	0.09	-0.18
B3LYP/aug-cc-pVTZ-J	4.10	0.87	3.83	0.08	0.19	0.15	3.81	0.71	3.47	0.01	0.09	-0.18
B972/aug-cc-pVTZ-J	3.87	0.77	3.62	0.06	0.17	0.17	3.61	0.63	3.28	-0.00	0.08	-0.15
B97D/aug-cc-pVTZ-J	3.92	0.86	3.63	0.06	0.17	0.16	3.67	0.73	3.32	0.00	0.08	-0.13
S55VWN5/aug-cc-pVTZ-J	3.77	0.63	3.48	0.07	0.17	0.18	3.49	0.50	3.13	0.00	0.09	-0.17
wB97X/aug-cc-pVTZ-J	3.86	0.94	3.56	0.07	0.16	0.15	3.62	0.80	3.24	0.00	0.07	-0.21
wB97XD/aug-cc-pVTZ-J	3.86	0.88	3.57	0.07	0.17	0.16	3.61	0.74	3.25	0.01	0.08	-0.18
$^3J_{H^\alpha C^{\gamma 2}}$												
SOPPA(CCSD)/6-311++Gee-J	3.75	0.46	3.44	-0.02	-0.16	1.12	3.47	0.37	3.25	-0.03	-0.13	0.66
B3LYP/6-311++Gee-J	4.11	0.70	3.77	-0.08	-0.12	1.28	3.82	0.59	3.59	-0.07	-0.12	0.79
B3LYP/aug-cc-pVTZ-J	4.12	0.67	3.77	-0.07	-0.12	1.28	3.82	0.55	3.59	-0.06	-0.13	0.80
B972/aug-cc-pVTZ-J	3.89	0.58	3.57	-0.07	-0.12	1.21	3.62	0.49	3.40	-0.06	-0.12	0.74
B97D/aug-cc-pVTZ-J	3.94	0.66	3.58	-0.09	-0.10	1.23	3.69	0.59	3.42	-0.06	-0.12	0.79
S55VWN5/aug-cc-pVTZ-J	3.80	0.47	3.45	-0.06	-0.12	1.16	3.50	0.37	3.26	-0.06	-0.11	0.67
wB97X/aug-cc-pVTZ-J	3.89	0.76	3.53	-0.05	-0.10	1.17	3.63	0.67	3.38	-0.06	-0.11	0.67
wB97XD/aug-cc-pVTZ-J	3.88	0.70	3.54	-0.06	-0.10	1.18	3.62	0.60	3.38	-0.06	-0.12	0.69
$^3J_{C' H^{\beta 3}}$												
SOPPA(CCSD)/6-311++Gee-J	3.65	-1.04	3.39	-0.01	0.31	-1.20	3.50	-1.32	3.34	-0.15	0.26	-0.64
B3LYP/6-311++Gee-J	3.94	-1.14	3.72	-0.11	0.30	-1.29	3.83	-1.38	3.70	-0.24	0.23	-0.69
B3LYP/aug-cc-pVTZ-J	3.90	-1.18	3.72	-0.10	0.30	-1.27	3.79	-1.42	3.69	-0.23	0.23	-0.68
B972/aug-cc-pVTZ-J	3.70	-1.13	3.51	-0.11	0.28	-1.22	3.58	-1.36	3.48	-0.23	0.22	-0.65
B97D/aug-cc-pVTZ-J	3.69	-1.17	3.45	-0.15	0.27	-1.22	3.59	-1.40	3.43	-0.26	0.21	-0.67
S55VWN5/aug-cc-pVTZ-J	3.77	-1.07	3.50	-0.06	0.28	-1.23	3.61	-1.29	3.44	-0.19	0.22	-0.64
wB97X/aug-cc-pVTZ-J	3.67	-0.96	3.46	-0.07	0.28	-1.18	3.56	-1.18	3.42	-0.20	0.25	-0.60
wB97XD/aug-cc-pVTZ-J	3.71	-1.00	3.50	-0.08	0.28	-1.21	3.60	-1.23	3.47	-0.21	0.24	-0.62
$^3J_{C' C^{\gamma 1}}$												
SOPPA(CCSD)/6-311++Gee-J	1.65	-0.79	1.29	-0.04	0.50	-0.90	1.59	-0.95	1.45	-0.18	0.39	-0.60
B3LYP/6-311++Gee-J	1.74	-0.81	1.37	-0.07	0.52	-0.98	1.73	-0.93	1.57	-0.22	0.39	-0.67
B3LYP/aug-cc-pVTZ-J	1.74	-0.81	1.37	-0.06	0.52	-0.97	1.72	-0.93	1.56	-0.21	0.39	-0.67
B972/aug-cc-pVTZ-J	1.77	-0.81	1.42	-0.07	0.51	-0.99	1.74	-0.94	1.59	-0.22	0.39	-0.67
B97D/aug-cc-pVTZ-J	1.89	-0.87	1.49	-0.09	0.56	-1.05	1.87	-1.00	1.69	-0.25	0.41	-0.75
S55VWN5/aug-cc-pVTZ-J	1.72	-0.81	1.37	-0.05	0.47	-0.95	1.68	-0.94	1.51	-0.20	0.37	-0.62
wB97X/aug-cc-pVTZ-J	1.82	-0.75	1.45	-0.06	0.51	-0.98	1.80	-0.87	1.64	-0.22	0.40	-0.63
wB97XD/aug-cc-pVTZ-J	1.78	-0.77	1.42	-0.07	0.51	-0.97	1.76	-0.89	1.61	-0.21	0.39	-0.64

Table S4: Fourier coeficients for Val calculated using the indicated method (continuation).

	α-conformer						β-conformer					
Method	C_0	C_1	C_2	C_3	S_1	S_2	C_0	C_1	C_2	C_3	S_1	S_2
$^3J_{C'C^{\gamma_2}}$												
SOPPA(CCSD)/6-311++Gee-J	1.69	-0.69	1.57	0.10	0.17	-0.47	1.60	-0.68	1.50	0.10	0.23	-0.20
B3LYP/6-311++Gee-J	1.79	-0.68	1.70	0.10	0.19	-0.49	1.73	-0.62	1.64	0.09	0.26	-0.20
B3LYP/aug-cc-pVTZ-J	1.78	-0.68	1.70	0.11	0.19	-0.49	1.73	-0.62	1.64	0.10	0.26	-0.20
B972/aug-cc-pVTZ-J	1.81	-0.68	1.74	0.09	0.20	-0.52	1.75	-0.64	1.67	0.08	0.27	-0.21
B97D/aug-cc-pVTZ-J	1.94	-0.72	1.85	0.09	0.22	-0.55	1.87	-0.67	1.77	0.08	0.30	-0.25
S55VWN5/aug-cc-pVTZ-J	1.75	-0.70	1.67	0.09	0.17	-0.50	1.68	-0.66	1.59	0.08	0.24	-0.19
wB97X/aug-cc-pVTZ-J	1.86	-0.63	1.78	0.09	0.20	-0.51	1.81	-0.57	1.71	0.09	0.28	-0.17
wB97XD/aug-cc-pVTZ-J	1.82	-0.65	1.74	0.09	0.19	-0.50	1.77	-0.59	1.68	0.09	0.26	-0.19
$^3J_{N'H^{\beta_3}}$												
SOPPA(CCSD)/6-311++Gee-J	-2.47	-0.32	-2.42	-0.14	0.10	-0.09	-2.15	0.27	-2.06	-0.04	-0.03	-0.13
B3LYP/6-311++Gee-J	-2.71	-0.60	-2.70	-0.18	0.12	-0.09	-2.28	0.21	-2.19	-0.02	-0.05	-0.14
B3LYP/aug-cc-pVTZ-J	-2.69	-0.59	-2.70	-0.19	0.12	-0.08	-2.26	0.23	-2.18	-0.03	-0.05	-0.13
B972/aug-cc-pVTZ-J	-2.57	-0.51	-2.58	-0.18	0.11	-0.08	-2.17	0.23	-2.09	-0.01	-0.05	-0.12
B97D/aug-cc-pVTZ-J	-2.53	-0.61	-2.54	-0.20	0.11	-0.06	-2.10	0.19	-2.01	-0.02	-0.06	-0.11
S55VWN5/aug-cc-pVTZ-J	-2.54	-0.39	-2.48	-0.14	0.10	-0.09	-2.17	0.27	-2.07	-0.01	-0.05	-0.13
wB97X/aug-cc-pVTZ-J	-2.56	-0.58	-2.51	-0.16	0.09	-0.10	-2.17	0.12	-2.06	-0.02	-0.05	-0.14
wB97XD/aug-cc-pVTZ-J	-2.55	-0.56	-2.52	-0.17	0.10	-0.09	-2.15	0.15	-2.05	-0.02	-0.05	-0.13
$^3J_{N'C^{\gamma_1}}$												
SOPPA(CCSD)/6-311++Gee-J	-1.17	-0.26	-1.21	-0.13	-0.08	0.11	-1.13	-0.02	-1.17	-0.10	-0.07	0.04
B3LYP/6-311++Gee-J	-1.18	-0.34	-1.22	-0.16	-0.08	0.14	-1.13	-0.07	-1.15	-0.11	-0.08	0.03
B3LYP/aug-cc-pVTZ-J	-1.17	-0.33	-1.22	-0.17	-0.08	0.14	-1.11	-0.07	-1.14	-0.11	-0.08	0.03
B972/aug-cc-pVTZ-J	-1.20	-0.33	-1.25	-0.16	-0.08	0.14	-1.14	-0.05	-1.17	-0.09	-0.08	0.03
B97D/aug-cc-pVTZ-J	-1.19	-0.31	-1.25	-0.17	-0.08	0.18	-1.13	-0.03	-1.16	-0.11	-0.09	0.03
S55VWN5/aug-cc-pVTZ-J	-1.23	-0.33	-1.27	-0.15	-0.08	0.10	-1.15	-0.04	-1.18	-0.08	-0.08	0.04
wB97X/aug-cc-pVTZ-J	-1.35	-0.48	-1.38	-0.16	-0.10	0.11	-1.24	-0.15	-1.26	-0.09	-0.08	0.02
wB97XD/aug-cc-pVTZ-J	-1.26	-0.41	-1.30	-0.16	-0.09	0.11	-1.17	-0.11	-1.20	-0.10	-0.08	0.03
$^3J_{N'C^{\gamma_2}}$												
SOPPA(CCSD)/6-311++Gee-J	-1.17	-0.30	-1.14	-0.10	0.20	-0.24	-1.15	-0.02	-1.12	-0.06	0.22	-0.31
B3LYP/6-311++Gee-J	-1.18	-0.38	-1.16	-0.14	0.20	-0.27	-1.14	-0.06	-1.09	-0.07	0.22	-0.35
B3LYP/aug-cc-pVTZ-J	-1.17	-0.37	-1.15	-0.14	0.19	-0.27	-1.13	-0.06	-1.08	-0.08	0.22	-0.35
B972/aug-cc-pVTZ-J	-1.21	-0.37	-1.19	-0.13	0.19	-0.27	-1.16	-0.04	-1.11	-0.06	0.21	-0.34
B97D/aug-cc-pVTZ-J	-1.19	-0.35	-1.19	-0.14	0.21	-0.26	-1.15	-0.01	-1.10	-0.07	0.23	-0.36
S55VWN5/aug-cc-pVTZ-J	-1.23	-0.37	-1.19	-0.12	0.19	-0.27	-1.17	-0.04	-1.13	-0.05	0.20	-0.32
wB97X/aug-cc-pVTZ-J	-1.36	-0.52	-1.31	-0.14	0.18	-0.30	-1.26	-0.15	-1.19	-0.06	0.20	-0.36
wB97XD/aug-cc-pVTZ-J	-1.27	-0.46	-1.24	-0.14	0.18	-0.29	-1.19	-0.11	-1.14	-0.06	0.20	-0.34

Table S5: Fourier coeficients for Leu calculated using the indicated method.

	α-conformer						β-conformer					
Method	C_0	C_1	C_2	C_3	S_1	S_2	C_0	C_1	C_2	C_3	S_1	S_2
$^3J_{H^\alpha H^{\beta 2}}$												
SOPPA(CCSD)/6-311++Gee-J	6.73	0.35	5.84	-0.19	-0.13	1.71	6.26	0.20	5.68	-0.19	0.05	0.73
B3LYP/6-311++Gee-J	7.41	0.45	6.51	-0.31	-0.05	2.00	6.93	0.29	6.40	-0.26	0.07	0.88
B3LYP/aug-cc-pVTZ-J	7.47	0.20	6.50	-0.32	-0.03	2.02	6.98	0.05	6.39	-0.27	0.08	0.89
B972/aug-cc-pVTZ-J	6.57	0.19	5.74	-0.29	-0.03	1.78	6.16	0.08	5.64	-0.25	0.06	0.78
B97D/aug-cc-pVTZ-J	6.19	0.28	5.31	-0.28	0.00	1.68	5.84	0.19	5.26	-0.23	0.06	0.77
S55VWN5/aug-cc-pVTZ-J	6.81	0.16	5.83	-0.25	-0.08	1.78	6.32	0.02	5.67	-0.23	0.06	0.73
wB97X/aug-cc-pVTZ-J	6.50	0.26	5.63	-0.25	-0.08	1.68	6.09	0.13	5.52	-0.23	0.04	0.69
wB97XD/aug-cc-pVTZ-J	6.61	0.27	5.74	-0.26	-0.06	1.73	6.19	0.13	5.63	-0.23	0.05	0.73
$^3J_{H^\alpha H^{\beta 3}}$												
SOPPA(CCSD)/6-311++Gee-J	6.66	0.43	5.73	-0.24	0.04	0.65	6.23	0.28	5.35	-0.22	0.17	-0.17
B3LYP/6-311++Gee-J	7.36	0.53	6.46	-0.36	0.03	0.81	6.92	0.39	6.06	-0.29	0.15	-0.16
B3LYP/aug-cc-pVTZ-J	7.42	0.29	6.46	-0.37	0.04	0.82	6.96	0.16	6.06	-0.29	0.15	-0.16
B972/aug-cc-pVTZ-J	6.53	0.27	5.70	-0.34	0.03	0.73	6.14	0.17	5.36	-0.26	0.13	-0.14
B97D/aug-cc-pVTZ-J	6.16	0.33	5.30	-0.33	0.02	0.72	5.83	0.26	5.00	-0.25	0.11	-0.08
S55VWN5/aug-cc-pVTZ-J	6.75	0.24	5.74	-0.30	0.05	0.71	6.30	0.10	5.36	-0.25	0.15	-0.18
wB97X/aug-cc-pVTZ-J	6.44	0.33	5.56	-0.30	0.05	0.62	6.07	0.21	5.21	-0.24	0.14	-0.23
wB97XD/aug-cc-pVTZ-J	6.55	0.34	5.67	-0.31	0.05	0.66	6.17	0.22	5.32	-0.25	0.14	-0.20
$^3J_{H^\alpha C^{\gamma 1}}$												
SOPPA(CCSD)/6-311++Gee-J	3.63	-0.62	3.56	-0.10	-0.06	0.62	3.34	-0.57	3.23	-0.05	-0.02	0.23
B3LYP/6-311++Gee-J	4.01	-0.58	3.99	-0.14	-0.06	0.70	3.70	-0.50	3.63	-0.07	-0.04	0.30
B3LYP/aug-cc-pVTZ-J	4.01	-0.62	3.99	-0.13	-0.06	0.70	3.70	-0.53	3.64	-0.07	-0.04	0.30
B972/aug-cc-pVTZ-J	3.81	-0.59	3.80	-0.13	-0.06	0.68	3.52	-0.52	3.47	-0.07	-0.04	0.29
B97D/aug-cc-pVTZ-J	3.86	-0.59	3.83	-0.15	-0.06	0.68	3.58	-0.49	3.51	-0.08	-0.05	0.32
S55VWN5/aug-cc-pVTZ-J	3.73	-0.58	3.64	-0.12	-0.05	0.67	3.43	-0.53	3.32	-0.07	-0.03	0.24
wB97X/aug-cc-pVTZ-J	3.73	-0.46	3.70	-0.11	-0.06	0.65	3.46	-0.40	3.39	-0.07	-0.03	0.23
wB97XD/aug-cc-pVTZ-J	3.78	-0.49	3.74	-0.11	-0.06	0.66	3.50	-0.44	3.43	-0.06	-0.04	0.25
$^3J_{C' H^{\beta 2}}$												
SOPPA(CCSD)/6-311++Gee-J	3.96	-1.04	3.66	-0.04	0.17	-1.07	3.79	-1.25	3.58	-0.03	0.13	-0.39
B3LYP/6-311++Gee-J	4.30	-1.12	4.05	-0.13	0.17	-1.18	4.17	-1.26	4.01	-0.09	0.13	-0.41
B3LYP/aug-cc-pVTZ-J	4.27	-1.17	4.05	-0.12	0.17	-1.16	4.14	-1.31	4.01	-0.09	0.12	-0.40
B972/aug-cc-pVTZ-J	4.03	-1.10	3.82	-0.14	0.18	-1.12	3.89	-1.25	3.77	-0.10	0.13	-0.39
B97D/aug-cc-pVTZ-J	4.00	-1.12	3.75	-0.16	0.19	-1.13	3.87	-1.25	3.72	-0.13	0.13	-0.43
S55VWN5/aug-cc-pVTZ-J	4.11	-1.07	3.82	-0.10	0.16	-1.12	3.93	-1.21	3.72	-0.07	0.12	-0.38
wB97X/aug-cc-pVTZ-J	4.01	-0.96	3.78	-0.10	0.15	-1.06	3.88	-1.09	3.72	-0.07	0.15	-0.33
wB97XD/aug-cc-pVTZ-J	4.05	-1.00	3.82	-0.10	0.16	-1.09	3.92	-1.14	3.77	-0.08	0.13	-0.35
$^3J_{C' H^{\beta 3}}$												
SOPPA(CCSD)/6-311++Gee-J	3.86	-1.20	3.36	-0.32	0.44	-1.65	3.79	-1.52	3.54	-0.40	0.25	-0.96
B3LYP/6-311++Gee-J	4.19	-1.29	3.70	-0.44	0.38	-1.81	4.18	-1.56	3.94	-0.50	0.15	-1.06
B3LYP/aug-cc-pVTZ-J	4.16	-1.34	3.71	-0.43	0.38	-1.80	4.15	-1.60	3.94	-0.49	0.16	-1.05
B972/aug-cc-pVTZ-J	3.92	-1.26	3.50	-0.41	0.35	-1.71	3.90	-1.51	3.71	-0.47	0.15	-1.00
B97D/aug-cc-pVTZ-J	3.90	-1.26	3.43	-0.44	0.32	-1.70	3.89	-1.51	3.65	-0.48	0.10	-1.01
S55VWN5/aug-cc-pVTZ-J	4.01	-1.22	3.49	-0.37	0.39	-1.71	3.93	-1.48	3.66	-0.44	0.19	-0.98
wB97X/aug-cc-pVTZ-J	3.91	-1.12	3.45	-0.38	0.38	-1.66	3.89	-1.38	3.66	-0.46	0.19	-0.94
wB97XD/aug-cc-pVTZ-J	3.94	-1.16	3.49	-0.39	0.37	-1.68	3.92	-1.42	3.70	-0.46	0.18	-0.97

Table S5: Fourier coeficients for Leu calculated using the indicated method (continuation).

	α-conformer						β-conformer					
Method	C_0	C_1	C_2	C_3	S_1	S_2	C_0	C_1	C_2	C_3	S_1	S_2
$^3J_{C'C^{\gamma_1}}$												
SOPPA(CCSD)/6-311++Gee-J	1.59	-0.79	1.43	-0.10	0.22	-0.59	1.48	-0.95	1.42	-0.10	0.17	-0.34
B3LYP/6-311++Gee-J	1.70	-0.77	1.55	-0.12	0.22	-0.65	1.62	-0.89	1.57	-0.11	0.16	-0.38
B3LYP/aug-cc-pVTZ-J	1.69	-0.77	1.55	-0.12	0.22	-0.64	1.61	-0.90	1.58	-0.11	0.16	-0.37
B972/aug-cc-pVTZ-J	1.72	-0.78	1.60	-0.12	0.24	-0.66	1.63	-0.92	1.61	-0.11	0.17	-0.38
B97D/aug-cc-pVTZ-J	1.83	-0.81	1.70	-0.14	0.25	-0.71	1.73	-0.95	1.71	-0.13	0.18	-0.43
S55VWN5/aug-cc-pVTZ-J	1.69	-0.81	1.54	-0.12	0.22	-0.65	1.60	-0.95	1.53	-0.11	0.16	-0.36
wB97X/aug-cc-pVTZ-J	1.76	-0.76	1.63	-0.12	0.23	-0.66	1.67	-0.89	1.63	-0.12	0.18	-0.35
wB97XD/aug-cc-pVTZ-J	1.74	-0.78	1.60	-0.12	0.23	-0.65	1.65	-0.90	1.62	-0.11	0.17	-0.36
$^3J_{N'H^{\beta_2}}$												
SOPPA(CCSD)/6-311++Gee-J	-2.98	0.16	-2.69	-0.04	0.32	-0.49	-2.55	0.63	-2.33	0.05	0.15	-0.52
B3LYP/6-311++Gee-J	-3.34	0.02	-3.04	-0.04	0.37	-0.57	-2.73	0.60	-2.49	0.08	0.13	-0.59
B3LYP/aug-cc-pVTZ-J	-3.32	0.03	-3.04	-0.05	0.37	-0.56	-2.71	0.61	-2.49	0.08	0.13	-0.58
B972/aug-cc-pVTZ-J	-3.14	0.04	-2.88	-0.05	0.34	-0.52	-2.58	0.57	-2.37	0.08	0.12	-0.53
B97D/aug-cc-pVTZ-J	-3.10	-0.01	-2.86	-0.06	0.34	-0.49	-2.51	0.54	-2.29	0.08	0.11	-0.53
S55VWN5/aug-cc-pVTZ-J	-3.09	0.10	-2.77	-0.03	0.32	-0.52	-2.59	0.60	-2.36	0.07	0.11	-0.53
wB97X/aug-cc-pVTZ-J	-3.11	-0.05	-2.80	-0.04	0.32	-0.53	-2.57	0.46	-2.33	0.07	0.12	-0.54
wB97XD/aug-cc-pVTZ-J	-3.10	-0.03	-2.81	-0.04	0.33	-0.53	-2.55	0.49	-2.33	0.07	0.12	-0.54
$^3J_{N'H^{\beta_3}}$												
SOPPA(CCSD)/6-311++Gee-J	-2.96	0.21	-2.90	0.02	-0.20	0.25	-2.58	0.67	-2.50	0.12	-0.19	0.07
B3LYP/6-311++Gee-J	-3.32	0.06	-3.28	-0.00	-0.22	0.31	-2.77	0.65	-2.70	0.18	-0.20	0.06
B3LYP/aug-cc-pVTZ-J	-3.31	0.07	-3.27	-0.01	-0.21	0.32	-2.75	0.66	-2.69	0.17	-0.20	0.06
B972/aug-cc-pVTZ-J	-3.12	0.08	-3.10	-0.01	-0.21	0.30	-2.62	0.63	-2.55	0.17	-0.19	0.07
B97D/aug-cc-pVTZ-J	-3.08	0.01	-3.07	-0.03	-0.20	0.32	-2.54	0.60	-2.47	0.17	-0.19	0.05
S55VWN5/aug-cc-pVTZ-J	-3.07	0.16	-3.00	0.02	-0.19	0.25	-2.62	0.65	-2.52	0.16	-0.19	0.07
wB97X/aug-cc-pVTZ-J	-3.10	-0.00	-3.04	0.02	-0.20	0.24	-2.61	0.52	-2.52	0.16	-0.19	0.06
wB97XD/aug-cc-pVTZ-J	-3.09	0.01	-3.04	0.00	-0.20	0.26	-2.59	0.55	-2.51	0.16	-0.19	0.05
$^3J_{N'C^{\gamma_1}}$												
SOPPA(CCSD)/6-311++Gee-J	-0.96	0.34	-1.04	-0.02	0.07	-0.04	-0.99	0.43	-1.07	0.01	0.08	-0.08
B3LYP/6-311++Gee-J	-0.97	0.30	-1.06	-0.04	0.07	-0.05	-0.99	0.38	-1.06	0.01	0.08	-0.10
B3LYP/aug-cc-pVTZ-J	-0.96	0.31	-1.06	-0.04	0.07	-0.05	-0.98	0.39	-1.05	0.01	0.08	-0.10
B972/aug-cc-pVTZ-J	-0.99	0.32	-1.11	-0.04	0.07	-0.05	-0.99	0.40	-1.09	0.01	0.08	-0.09
B97D/aug-cc-pVTZ-J	-0.97	0.34	-1.10	-0.04	0.07	-0.03	-0.98	0.42	-1.08	0.01	0.08	-0.09
S55VWN5/aug-cc-pVTZ-J	-1.03	0.32	-1.13	-0.03	0.07	-0.07	-1.03	0.42	-1.11	0.01	0.07	-0.10
wB97X/aug-cc-pVTZ-J	-1.10	0.29	-1.21	-0.04	0.07	-0.07	-1.07	0.37	-1.16	0.01	0.08	-0.10
wB97XD/aug-cc-pVTZ-J	-1.04	0.29	-1.15	-0.04	0.07	-0.06	-1.03	0.38	-1.12	0.01	0.08	-0.10

Table S6: Fourier coeficients for Ile calculated using the indicated method.

	α-conformer						β-conformer					
Method	C_0	C_1	C_2	C_3	S_1	S_2	C_0	C_1	C_2	C_3	S_1	S_2
$^3J_{H^\alpha H^{\beta 3}}$												
SOPPA(CCSD)/6-311++G**-J	6.36	1.00	5.54	0.07	-0.14	1.01	5.93	0.66	5.20	-0.09	0.03	0.21
B3LYP/6-311++Gee-J	6.93	1.17	6.15	-0.04	-0.10	1.20	6.50	0.81	5.81	-0.17	0.03	0.26
B3LYP/aug-cc-pVTZ-J	6.95	0.92	6.13	-0.05	-0.09	1.22	6.51	0.56	5.80	-0.17	0.03	0.27
B972/aug-cc-pVTZ-J	6.14	0.80	5.43	-0.07	-0.08	1.08	5.76	0.51	5.14	-0.17	0.02	0.24
B97D/aug-cc-pVTZ-J	5.78	0.86	5.02	-0.09	-0.06	1.03	5.46	0.60	4.77	-0.16	0.02	0.26
S55VWN5/aug-cc-pVTZ-J	6.40	0.76	5.50	-0.00	-0.10	1.07	5.95	0.43	5.16	-0.14	0.03	0.20
wB97X/aug-cc-pVTZ-J	6.08	0.85	5.30	-0.01	-0.10	0.99	5.70	0.56	5.00	-0.14	0.01	0.16
wB97XD/aug-cc-pVTZ-J	6.18	0.88	5.40	-0.03	-0.09	1.03	5.80	0.56	5.11	-0.15	0.01	0.19
$^3J_{H^\alpha C^{\gamma 1}}$												
SOPPA(CCSD)/6-311++G**-J	3.65	0.25	3.30	0.08	-0.26	1.12	3.36	0.14	3.11	0.03	-0.15	0.73
B3LYP/6-311++Gee-J	3.98	0.44	3.62	0.04	-0.24	1.27	3.68	0.31	3.43	-0.00	-0.15	0.86
B3LYP/aug-cc-pVTZ-J	3.98	0.40	3.62	0.05	-0.25	1.27	3.68	0.28	3.43	0.01	-0.16	0.86
B972/aug-cc-pVTZ-J	3.77	0.35	3.45	0.03	-0.24	1.20	3.49	0.24	3.26	0.01	-0.15	0.81
B97D/aug-cc-pVTZ-J	3.80	0.40	3.44	0.02	-0.23	1.21	3.54	0.31	3.27	0.01	-0.15	0.85
S55VWN5/aug-cc-pVTZ-J	3.71	0.27	3.34	0.04	-0.23	1.17	3.41	0.14	3.14	0.00	-0.14	0.74
wB97X/aug-cc-pVTZ-J	3.75	0.50	3.40	0.05	-0.22	1.17	3.49	0.38	3.23	0.00	-0.14	0.75
wB97XD/aug-cc-pVTZ-J	3.77	0.46	3.42	0.04	-0.22	1.18	3.50	0.34	3.25	0.01	-0.14	0.77
$^3J_{H^\alpha C^{\gamma 2}}$												
SOPPA(CCSD)/6-311++G**-J	3.80	0.71	3.53	0.14	0.12	0.09	3.53	0.60	3.09	0.07	-0.02	-0.21
B3LYP/6-311++Gee-J	4.14	0.97	3.90	0.10	0.10	0.07	3.88	0.84	3.43	0.05	-0.07	-0.25
B3LYP/aug-cc-pVTZ-J	4.15	0.94	3.91	0.11	0.10	0.08	3.88	0.80	3.44	0.06	-0.07	-0.25
B972/aug-cc-pVTZ-J	3.92	0.83	3.69	0.09	0.09	0.11	3.68	0.72	3.26	0.04	-0.06	-0.22
B97D/aug-cc-pVTZ-J	3.97	0.92	3.70	0.08	0.10	0.09	3.74	0.82	3.29	0.04	-0.07	-0.20
S55VWN5/aug-cc-pVTZ-J	3.82	0.70	3.54	0.10	0.10	0.12	3.56	0.59	3.10	0.04	-0.04	-0.23
wB97X/aug-cc-pVTZ-J	3.92	1.00	3.63	0.10	0.08	0.09	3.69	0.88	3.22	0.05	-0.07	-0.27
wB97XD/aug-cc-pVTZ-J	3.91	0.95	3.65	0.10	0.09	0.10	3.68	0.83	3.23	0.05	-0.06	-0.25
$^3J_{C' H^{\beta 3}}$												
SOPPA(CCSD)/6-311++G**-J	3.71	-0.98	3.40	-0.12	0.41	-1.27	3.57	-1.34	3.42	-0.25	0.29	-0.66
B3LYP/6-311++Gee-J	3.99	-1.08	3.73	-0.24	0.40	-1.35	3.91	-1.41	3.77	-0.34	0.25	-0.72
B3LYP/aug-cc-pVTZ-J	3.96	-1.13	3.73	-0.24	0.40	-1.34	3.87	-1.44	3.77	-0.33	0.25	-0.70
B972/aug-cc-pVTZ-J	3.75	-1.07	3.52	-0.23	0.38	-1.28	3.66	-1.37	3.54	-0.32	0.24	-0.68
B97D/aug-cc-pVTZ-J	3.74	-1.12	3.46	-0.28	0.37	-1.27	3.66	-1.41	3.49	-0.36	0.22	-0.69
S55VWN5/aug-cc-pVTZ-J	3.83	-1.01	3.51	-0.18	0.37	-1.29	3.68	-1.31	3.50	-0.28	0.24	-0.66
wB97X/aug-cc-pVTZ-J	3.73	-0.90	3.47	-0.19	0.37	-1.23	3.63	-1.19	3.50	-0.30	0.27	-0.61
wB97XD/aug-cc-pVTZ-J	3.76	-0.94	3.52	-0.20	0.37	-1.26	3.67	-1.24	3.55	-0.30	0.25	-0.64
$^3J_{C' C^{\gamma 1}}$												
SOPPA(CCSD)/6-311++G**-J	1.61	-0.70	1.51	0.08	0.20	-0.38	1.48	-0.70	1.39	0.02	0.22	-0.12
B3LYP/6-311++Gee-J	1.70	-0.67	1.64	0.07	0.21	-0.40	1.61	-0.63	1.53	0.01	0.25	-0.11
B3LYP/aug-cc-pVTZ-J	1.69	-0.67	1.64	0.08	0.21	-0.40	1.60	-0.63	1.53	0.02	0.25	-0.11
B972/aug-cc-pVTZ-J	1.72	-0.69	1.68	0.06	0.23	-0.42	1.62	-0.66	1.56	0.00	0.26	-0.11
B97D/aug-cc-pVTZ-J	1.84	-0.73	1.78	0.06	0.24	-0.45	1.72	-0.68	1.65	-0.01	0.28	-0.14
S55VWN5/aug-cc-pVTZ-J	1.69	-0.72	1.62	0.06	0.21	-0.41	1.57	-0.69	1.49	0.01	0.23	-0.10
wB97X/aug-cc-pVTZ-J	1.77	-0.65	1.72	0.06	0.22	-0.41	1.67	-0.60	1.59	0.01	0.26	-0.07
wB97XD/aug-cc-pVTZ-J	1.74	-0.67	1.69	0.06	0.21	-0.41	1.64	-0.62	1.57	0.01	0.25	-0.09

Table S6: Fourier coeficients for Ile calculated using the indicated method (continuation).

	α-conformer						β-conformer					
Method	C_0	C_1	C_2	C_3	S_1	S_2	C_0	C_1	C_2	C_3	S_1	S_2
$^3J_{C'C^{\gamma_2}}$												
SOPPA(CCSD)/6-311++G**-J	1.61	-0.79	1.38	-0.23	0.60	-1.07	1.56	-0.96	1.54	-0.29	0.40	-0.72
B3LYP/6-311++Gee-J	1.70	-0.79	1.47	-0.28	0.61	-1.16	1.69	-0.93	1.67	-0.33	0.40	-0.80
B3LYP/aug-cc-pVTZ-J	1.69	-0.80	1.47	-0.27	0.61	-1.15	1.68	-0.94	1.66	-0.32	0.40	-0.79
B972/aug-cc-pVTZ-J	1.72	-0.80	1.52	-0.28	0.61	-1.17	1.71	-0.95	1.69	-0.32	0.40	-0.80
B97D/aug-cc-pVTZ-J	1.84	-0.86	1.60	-0.32	0.66	-1.25	1.83	-1.01	1.79	-0.36	0.42	-0.88
S55VWN5/aug-cc-pVTZ-J	1.68	-0.80	1.46	-0.24	0.56	-1.12	1.64	-0.95	1.61	-0.30	0.38	-0.74
wB97X/aug-cc-pVTZ-J	1.78	-0.75	1.55	-0.27	0.61	-1.16	1.76	-0.89	1.74	-0.33	0.41	-0.76
wB97XD/aug-cc-pVTZ-J	1.74	-0.76	1.52	-0.28	0.60	-1.15	1.72	-0.90	1.71	-0.33	0.40	-0.77
$^3J_{N'H^{\beta_3}}$												
SOPPA(CCSD)/6-311++G**-J	-2.56	-0.22	-2.50	-0.11	0.04	-0.13	-2.22	0.38	-2.13	-0.03	0.01	-0.18
B3LYP/6-311++Gee-J	-2.81	-0.46	-2.81	-0.14	0.03	-0.13	-2.35	0.33	-2.26	-0.00	0.00	-0.20
B3LYP/aug-cc-pVTZ-J	-2.80	-0.45	-2.81	-0.15	0.04	-0.12	-2.33	0.35	-2.25	-0.01	0.00	-0.20
B972/aug-cc-pVTZ-J	-2.66	-0.39	-2.67	-0.14	0.03	-0.11	-2.24	0.34	-2.16	0.00	0.00	-0.18
B97D/aug-cc-pVTZ-J	-2.63	-0.48	-2.65	-0.16	0.03	-0.09	-2.17	0.30	-2.08	-0.00	-0.01	-0.17
S55VWN5/aug-cc-pVTZ-J	-2.63	-0.28	-2.57	-0.11	0.03	-0.14	-2.23	0.37	-2.13	0.01	-0.00	-0.18
wB97X/aug-cc-pVTZ-J	-2.65	-0.46	-2.59	-0.12	0.03	-0.15	-2.23	0.22	-2.12	-0.01	0.00	-0.19
wB97XD/aug-cc-pVTZ-J	-2.64	-0.45	-2.61	-0.13	0.03	-0.14	-2.21	0.26	-2.11	-0.00	0.00	-0.19
$^3J_{N'C^{\gamma_1}}$												
SOPPA(CCSD)/6-311++G**-J	-1.02	-0.09	-0.97	-0.11	0.20	-0.27	-1.02	0.09	-1.01	-0.09	0.21	-0.31
B3LYP/6-311++Gee-J	-1.02	-0.15	-0.98	-0.14	0.20	-0.29	-1.01	0.05	-0.98	-0.10	0.21	-0.34
B3LYP/aug-cc-pVTZ-J	-1.01	-0.15	-0.97	-0.14	0.19	-0.29	-1.00	0.06	-0.97	-0.10	0.21	-0.34
B972/aug-cc-pVTZ-J	-1.05	-0.14	-1.01	-0.14	0.20	-0.29	-1.03	0.07	-1.00	-0.09	0.21	-0.34
B97D/aug-cc-pVTZ-J	-1.02	-0.12	-1.01	-0.14	0.21	-0.28	-1.01	0.10	-0.99	-0.10	0.23	-0.35
S55VWN5/aug-cc-pVTZ-J	-1.08	-0.14	-1.02	-0.13	0.19	-0.30	-1.05	0.07	-1.02	-0.08	0.20	-0.33
wB97X/aug-cc-pVTZ-J	-1.17	-0.25	-1.11	-0.14	0.19	-0.33	-1.12	-0.01	-1.07	-0.09	0.20	-0.36
wB97XD/aug-cc-pVTZ-J	-1.10	-0.21	-1.05	-0.14	0.19	-0.31	-1.06	0.01	-1.03	-0.09	0.20	-0.34
$^3J_{N'C^{\gamma_2}}$												
SOPPA(CCSD)/6-311++G**-J	-1.22	-0.27	-1.24	-0.06	-0.10	0.11	-1.17	-0.02	-1.20	-0.06	-0.09	0.01
B3LYP/6-311++Gee-J	-1.24	-0.34	-1.26	-0.09	-0.10	0.15	-1.16	-0.07	-1.18	-0.06	-0.09	0.00
B3LYP/aug-cc-pVTZ-J	-1.23	-0.33	-1.25	-0.09	-0.10	0.15	-1.15	-0.06	-1.17	-0.06	-0.09	0.00
B972/aug-cc-pVTZ-J	-1.26	-0.34	-1.28	-0.08	-0.10	0.15	-1.18	-0.05	-1.19	-0.05	-0.09	0.00
B97D/aug-cc-pVTZ-J	-1.24	-0.30	-1.28	-0.08	-0.10	0.19	-1.17	-0.02	-1.18	-0.05	-0.10	-0.01
S55VWN5/aug-cc-pVTZ-J	-1.28	-0.34	-1.30	-0.07	-0.10	0.11	-1.19	-0.04	-1.20	-0.04	-0.09	0.01
wB97X/aug-cc-pVTZ-J	-1.41	-0.48	-1.42	-0.08	-0.12	0.10	-1.28	-0.15	-1.28	-0.04	-0.09	-0.01
wB97XD/aug-cc-pVTZ-J	-1.32	-0.42	-1.34	-0.08	-0.11	0.12	-1.21	-0.11	-1.23	-0.05	-0.09	-0.00

Table S7: Fourier coefficient for the indicated AAs and SSCCs obtained empirically by Schmidt *et al.*[a] and Pérez *et al.*[b].

	Schmidt				Pérez		
SSCC	C_0	C_1	C_2	S_1	C_0	C_1	C_2
**Valine**							
$^3J_{H^\alpha H^\beta}$	5.35	-1.86	3.81	-0.37	5.40	-1.37	3.61
$^3J_{H^\alpha C^{\gamma_1,\gamma_2}}$	3.35	-1.58	2.46	0.10	3.26	-0.96	2.67
$^3J_{C'H^\beta}$	2.99	-1.99	2.48	-0.59	3.11	-1.58	2.01
$^3J_{C'C^{\gamma_1,\gamma_2}}$	1.66	-1.11	1.11	0.10	1.61	-0.87	1.15
$^3J_{N'H^\beta}$	-1.99	0.93	-1.26	-0.17	-2.08	0.75	-1.15
$^3J_{N'C^{\gamma_1,\gamma_2}}$	-1.03	0.55	-0.68	-0.02	-0.96	0.49	-0.65
**Leucine**							
$^3J_{H^\alpha H^{\beta_2,\beta_3}}$	6.04	-1.86	3.81	-0.37	6.01	-1.37	3.61
$^3J_{H^\alpha C^\gamma}$	3.31	-1.58	2.46	0.10	3.41	-0.96	2.67
$^3J_{C'H^{\beta_2,\beta_3}}$	3.33	-1.99	2.48	-0.59	3.41	-1.58	2.01
$^3J_{C'C^\gamma}$	1.64	-1.11	1.11	0.10	1.67	-0.87	1.15
$^3J_{N'H^{\beta_2,\beta_3}}$	-2.21	0.93	-1.26	-0.17	-2.28	0.75	-1.15
$^3J_{N'C^\gamma}$	-1.02	0.55	-0.68	-0.02	-1.01	0.49	-0.65
**Isoleucine**							
$^3J_{H^\alpha H^\beta}$	5.38	-1.86	3.81	-0.37	5.40	-1.37	3.61
$^3J_{H^\alpha C^{\gamma_1,\gamma_2}}$	3.17	-1.58	2.46	0.10	3.18	-0.96	2.67
$^3J_{C'H^\beta}$	3.01	-1.99	2.48	-0.59	3.11	-1.58	2.01
$^3J_{C'C^{\gamma_1,\gamma_2}}$	1.57	-1.11	1.11	0.10	1.57	-0.87	1.15
$^3J_{N'H^\beta}$	-2.00	0.93	-1.26	-0.17	-2.08	0.75	-1.15
$^3J_{N'C^{\gamma_1,\gamma_2}}$	-0.97	0.55	-0.68	-0.02	-0.93	0.49	-0.65

[a] J. M. Schmidt, Y. Hua, and F. Löhr. Asymmetric Karplus curves for the protein side-chain ^{3}J couplings. J. Biomol. NMR, 37:287–301, 2007.

[b] C. Pérez, F. Löhr, H. Rüterjans, and J. M. Schmidt. Self-consistent Karplus parametrization of ^{3}J coupling depending on the polypeptide side-chain torsion χ_1. J. Am. Chem. Soc., 123:7081–7093, 2001.

Table S8: Rmsd (Hz) and awrmsd (%) for Val, Leu and Ile when compare: a) results from aug-cc-pVTZ-J and 6-311++G**-J basis sets at B3LYP level; b) results for α and β conformers calculated at SOPPA-CCSD/6-311++G**-J; and c) results for the three staggered χ_2 conformers and the average ones at SOPPA-CCSD/6-311++G**-J. For a) and c) only the α conformer is considered.

**Valine**	$^3J_{H^\alpha H^\beta}$	$^3J_{H^\alpha C^{\gamma 1}}$	$^3J_{H^\alpha C^{\gamma 2}}$	$^3J_{C' H^\beta}$	$^3J_{C' C^{\gamma 1}}$	$^3J_{C' C^{\gamma 2}}$	$^3J_{N' H^\beta}$	$^3J_{N' C^{\gamma 1}}$	$^3J_{N' C^{\gamma 2}}$	awrmsd (%)
aug-cc-pVTZ-J vs 6-311++G**-J	0.18	0.03	0.02	0.05	0.01	0.01	0.02	0.01	0.02	1.2
α $v.s.$ β^a	0.76	0.44	0.45	0.48	0.30	0.22	0.60	0.18	0.21	15.4
α v.s Schmidt	2.24	1.36	1.48	1.47	0.74	0.49	1.24	0.71	0.73	44.3
α v.s Pérez	2.46	1.77	1.84	1.56	0.74	0.56	1.32	0.71	0.74	48.2
β v.s Schmidt	1.75	1.10	1.15	1.15	0.57	0.36	0.74	0.55	0.59	35.7
β v.s Pérez	2.01	1.55	1.57	1.20	0.56	0.45	0.76	0.55	0.59	39.8
**Leucine**	$^3J_{H^\alpha H^{\beta_1}}$	$^3J_{H^\alpha H^{\beta_2}}$	$^3J_{H^\alpha C^{\gamma 1}}$	$^3J_{C' H^{\beta_1}}$	$^3J_{C' C^{\beta_2}}$	$^3J_{C' C^{\gamma 1}}$	$^3J_{N' H^{\beta_1}}$	$^3J_{N' C^{\beta_2}}$	$^3J_{N' C^{\gamma 1}}$	rmsd%
aug-cc-pVTZ-J vs 6-311++G**-J	0.19	0.18	0.03	0.05	0.05	0.01	0.02	0.02	0.01	1.3
α $v.s.$ β^a	0.86	0.78	0.47	0.53	0.58	0.24	0.62	0.59	0.08	14.5
α v.s Schmidt	2.44	2.13	0.84	1.55	1.64	0.50	1.42	1.48	0.31	37.1
α v.s Pérez	2.55	2.27	1.17	1.55	1.71	0.54	1.47	1.48	0.31	39.2
β v.s Schmidt	1.93	1.73	0.52	1.23	1.37	0.39	0.96	1.02	0.31	30.8
β v.s Pérez	2.07	1.92	0.92	1.19	1.33	0.39	0.96	0.97	0.30	32.3
average vs 60° conf.	0.42	0.14	0.24	0.18	0.17	0.14	0.15	0.20	0.14	6.4
average vs 180° conf.	0.31	0.19	0.16	0.13	0.18	0.15	0.09	0.27	0.16	6.3
average vs -60° conf.	0.17	0.13	0.32	0.13	0.10	0.08	0.08	0.15	0.18	5.5
**Isoleucine**	$^3J_{H^\alpha H^\beta}$	$^3J_{H^\alpha C^{\gamma 1}}$	$^3J_{H^\alpha C^{\gamma 2}}$	$^3J_{C' H^\beta}$	$^3J_{C' C^{\gamma 1}}$	$^3J_{C' C^{\gamma 2}}$	$^3J_{N' H^\beta}$	$^3J_{C' C^{\gamma 1}}$	$^3J_{C' C^{\gamma 2}}$	rmsd%
aug-cc-pVTZ-J vs 6-311++G**-J	0.18	0.03	0.03	0.05	0.01	0.02	0.02	0.01	0.01	1.2
α $v.s.$ β^a	0.80	0.44	0.48	0.54	0.24	0.34	0.51	0.13	0.20	15.7
α v.s Schmidt	2.47	1.35	1.47	1.55	0.42	0.90	1.28	0.54	0.75	44.7
α v.s Pérez	2.66	1.72	1.90	1.65	0.50	0.90	1.33	0.56	0.75	48.8
β v.s Schmidt	1.91	1.01	1.21	1.24	0.29	0.68	0.76	0.48	0.59	36.1
β v.s Pérez	2.13	1.42	1.65	1.27	0.38	0.67	0.78	0.49	0.58	40.3
average vs 60° conf.	0.27	0.17	0.13	0.11	0.16	0.12	0.12	0.16	0.14	7.1
average vs 180° conf.	0.11	0.30	0.08	0.07	0.08	0.08	0.10	0.15	0.06	5.3
average vs -60° conf.	0.37	0.20	0.09	0.13	0.13	0.13	0.20	0.10	0.10	6.6

[a] Comparizon between the SOPPA-CCSD/6-311++G**-J results between α and β conformers.

Table S9: Rmsd (Hz) and rmsd% for Valine, Leucine and Isoleucine when compare with SOPPA-CCSD/6-311++G**-J results. Only α-conformer results are shown.

Method	$^3J_{H^\alpha H^\beta}$	$^3J_{H^\alpha C^{\gamma 1}}$	$^3J_{H^\alpha C^{\gamma 2}}$	$^3J_{C' H^\beta}$	$^3J_{C' C^{\gamma 1}}$	$^3J_{C' C^{\gamma 2}}$	$^3J_{N' H^\beta}$	$^3J_{N' C^{\gamma 1}}$	$^3J_{N' C^{\gamma 2}}$	rmsd%
Valine										
B3LYP/6-311++G**-J	0.73	0.47	0.48	0.39	0.12	0.14	0.37	0.07	0.07	10
B3LYP/aug-cc-pVTZ-J	0.75	0.48	0.48	0.37	0.12	0.13	0.35	0.06	0.06	10
B972/aug-cc-pVTZ-J	0.25	0.21	0.20	0.14	0.17	0.18	0.20	0.07	0.08	7
B97D/aug-cc-pVTZ-J	0.64	0.28	0.28	0.15	0.31	0.33	0.23	0.08	0.06	10
S55VWN5/aug-cc-pVTZ-J	0.19	0.05	0.07	0.15	0.10	0.10	0.10	0.09	0.09	5
wB97X/aug-cc-pVTZ-J	0.31	0.26	0.27	0.09	0.21	0.23	0.22	0.27	0.28	12
wB97XD/aug-cc-pVTZ-J	0.20	0.23	0.23	0.12	0.17	0.18	0.20	0.15	0.17	8
	$^3J_{H^\alpha H^{\beta 1}}$	$^3J_{H^\alpha H^{\beta 2}}$	$^3J_{H^\alpha C^{\gamma 1}}$	$^3J_{C' H^{\beta 1}}$	$^3J_{C' C^{\beta 2}}$	$^3J_{C' C^{\gamma 1}}$	$^3J_{N' H^{\beta 1}}$	$^3J_{N' C^{\beta 2}}$	$^3J_{N' C^{\gamma 1}}$	rmsd%
Leucine										
B3LYP/6-311++G**-J	0.86	0.88	0.49	0.45	0.44	0.15	0.45	0.46	0.04	12
B3LYP/aug-cc-pVTZ-J	0.92	0.94	0.49	0.43	0.42	0.14	0.43	0.45	0.03	12
B972/aug-cc-pVTZ-J	0.24	0.20	0.25	0.16	0.16	0.19	0.23	0.24	0.06	6
B97D/aug-cc-pVTZ-J	0.67	0.60	0.30	0.13	0.15	0.32	0.21	0.23	0.05	8
S55VWN5/aug-cc-pVTZ-J	0.17	0.17	0.12	0.20	0.19	0.14	0.13	0.14	0.10	5
wB97X/aug-cc-pVTZ-J	0.29	0.26	0.18	0.12	0.12	0.23	0.21	0.23	0.19	8
wB97XD/aug-cc-pVTZ-J	0.17	0.14	0.22	0.15	0.14	0.20	0.20	0.22	0.12	6
	$^3J_{H^\alpha H^\beta}$	$^3J_{H^\alpha C^{\gamma 1}}$	$^3J_{H^\alpha C^{\gamma 2}}$	$^3J_{C' H^\beta}$	$^3J_{C' C^{\gamma 1}}$	$^3J_{C' C^{\gamma 2}}$	$^3J_{N' H^\beta}$	$^3J_{N' C^{\gamma 1}}$	$^3J_{N' C^{\gamma 2}}$	rmsd%
Isoleucine										
B3LYP/6-311++G**-J	0.74	0.44	0.47	0.39	0.13	0.13	0.37	0.05	0.07	10
B3LYP/aug-cc-pVTZ-J	0.75	0.43	0.47	0.37	0.12	0.12	0.36	0.05	0.06	9
B972/aug-cc-pVTZ-J	0.30	0.19	0.19	0.14	0.17	0.17	0.20	0.06	0.08	7
B97D/aug-cc-pVTZ-J	0.71	0.22	0.26	0.16	0.31	0.32	0.23	0.04	0.07	10
S55VWN5/aug-cc-pVTZ-J	0.19	0.08	0.04	0.15	0.12	0.10	0.10	0.08	0.09	5
wB97X/aug-cc-pVTZ-J	0.35	0.22	0.25	0.10	0.22	0.22	0.20	0.22	0.27	11
wB97XD/aug-cc-pVTZ-J	0.24	0.22	0.22	0.12	0.19	0.18	0.20	0.13	0.16	8

[a] Comparizon between the SOPPA-CCSD/6-311++G**-J results between α and β conformers.

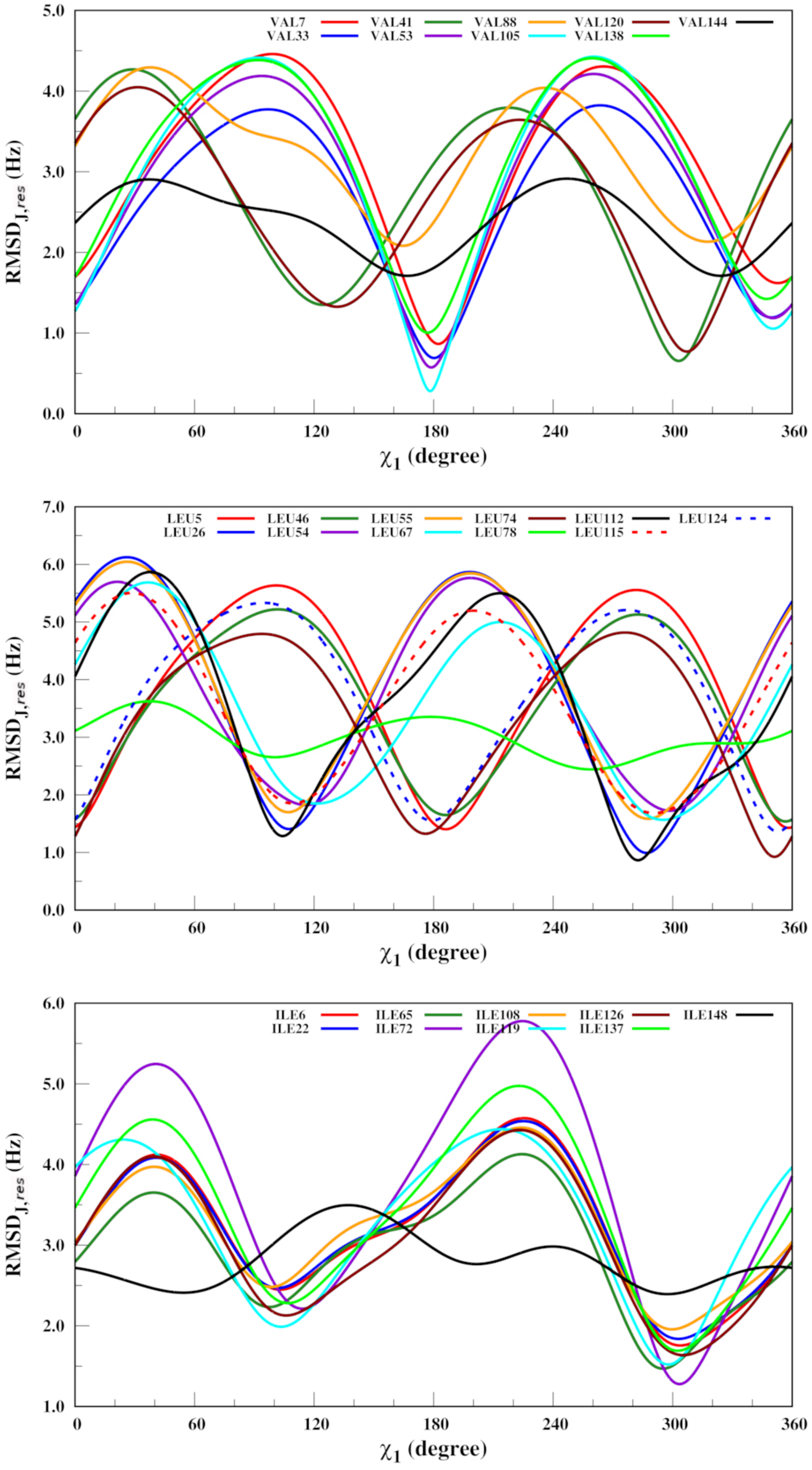

Figure S1: RMSD$_{J,res}$ for all residues of the three amino acids.

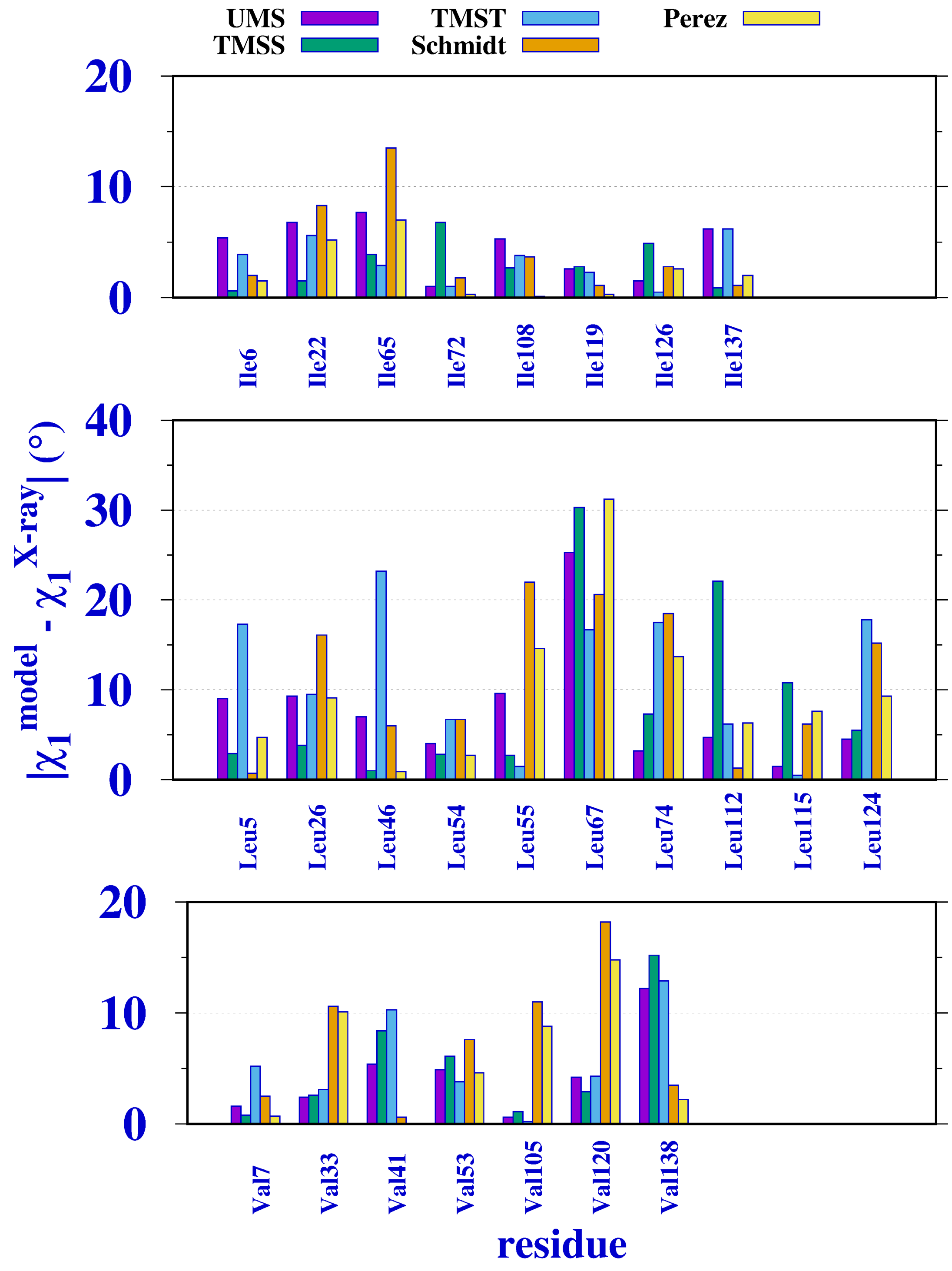

Figure S2: Deviations between χ_1 angles (degree) calculated with the indicated model and those of X-ray average ones.

Table S10: $RMSD_{\chi_1}^{X-ray}$ values (rmsd against the χ_1 angles calculated with the indicated method and those obtained as average X-ray). Superindex correspond to the maximum deviation.

Method	Val	Leu	Ile	All
UMS	5.7^{12}	10.1^{25}	5.1^{8}	7.6^{25}
TSS	7.1^{15}	12.8^{30}	3.6^{7}	9.2^{30}
TST	7.0^{13}	13.8^{23}	3.8^{6}	9.7^{23}
Schmidt	9.6^{18}	13.6^{22}	5.9^{14}	10.6^{22}
Pérez	7.8^{15}	12.9^{31}	3.3^{7}	9.3^{31}
n[a]	7	10	8	25

[a] Number of values.
